# T-Cell Immunoglobulin and Mucin Domain 1 (TIM-1) Is a Functional Entry Factor for Tick-Borne Encephalitis Virus

**DOI:** 10.1128/mbio.02860-21

**Published:** 2022-01-25

**Authors:** Xiaowei Zhang, Cuiqin Liang, Hanzhong Wang, Zhengyuan Guo, Heng Rong, Jingdi Pan, Wei Li, Rongjuan Pei, Xinwen Chen, Zhiping Zhang, Xian-En Zhang, Zongqiang Cui

**Affiliations:** a State Key Laboratory of Virology, Wuhan Institute of Virology, Center for Biosafety Mega-Science, Chinese Academy of Sciences, Wuhan, China; b Key Laboratory of Special Pathogens and Biosafety, Center for Emerging Infectious Diseases, Wuhan Institute of Virology, Chinese Academy of Sciences, Wuhan, China; c University of Chinese Academy of Sciences, Beijing, China; d National Laboratory of Biomacromolecules, CAS Center for Excellence in Biomacromolecules, Institute of Biophysics, Chinese Academy of Sciences, Beijing China; Washington University School of Medicine

**Keywords:** tick-borne encephalitis virus (TBEV), T-cell immunoglobulin and mucin domain 1 (TIM-1), virus entry

## Abstract

Tick-borne encephalitis virus (TBEV) is the causative agent of a potentially fatal neurological infection affecting humans. The host factors required for viral entry have yet to be described. Here, we found that T-cell immunoglobulin and mucin domain 1 (TIM-1) acted as the cellular entry factor for TBEV. Using a virus overlay protein binding assay, TIM-1 was identified as a virion-interacting protein. Cells that were relatively resistant to TBEV infection became highly susceptible to infection when TIM-1 was ectopically expressed. TIM-1 knockout and viral RNA bypass assays showed that TIM-1 functioned in the entry phase of TBEV infection. TIM-1 mediated TBEV uptake and was cointernalized with virus particles into the cell. Antibodies for TIM-1, soluble TIM-1, or TIM-1 knockdown significantly inhibited TBEV infection in permissive cells. Furthermore, in TIM-1 knockout mice, TIM-1 deficiency markedly lowered viral burden and reduced mortality and morbidity, highlighting the functional relevance of TIM-1 *in vivo*. With TIM-1, we have identified a key host factor for TBEV entry and a potential target for antiviral intervention.

## INTRODUCTION

Tick-borne encephalitis virus (TBEV), a neurotropic virus transmitted by ticks, is a member of the genus *Flavivirus*, within the *Flaviviridae* family. In humans, TBEV can cause biphasic febrile illness that may progress to neurological complications such as meningitis, encephalitis, or myelitis, leading to severe long-lasting neurological sequelae and sometimes death (1–3). There are no specific and effective therapies available for TBEV, and despite vaccines against the virus, it remains one of the main etiological agents of central nervous system infections in Europe and Northeast Asia. More than 13,000 clinical cases of tick-borne encephalitis occur annually, with increased numbers over the past few decades (4, 5).

The life cycle of TBEV begins with the attachment of the virions to receptors on the host cell surface membrane, which subsequently leads to receptor-mediated endocytosis (6). The process of TBEV entry into a target cell uses host molecules which act as entry factors or cellular receptors. Though a few cell surface molecules have been suggested to play a role in virion attachment (7, 8), the molecular interactions mediating TBEV entry are poorly understood, and the host factors involved in TBEV entry have yet to be identified and characterized. In this study, we show that TBEV uses T-cell immunoglobulin and mucin domain 1 (TIM-1) as a cellular entry factor and that this interaction can form a productive infection.

## RESULTS

### TIM-1 is identified as a TBEV-associated protein.

**FIG 1:**
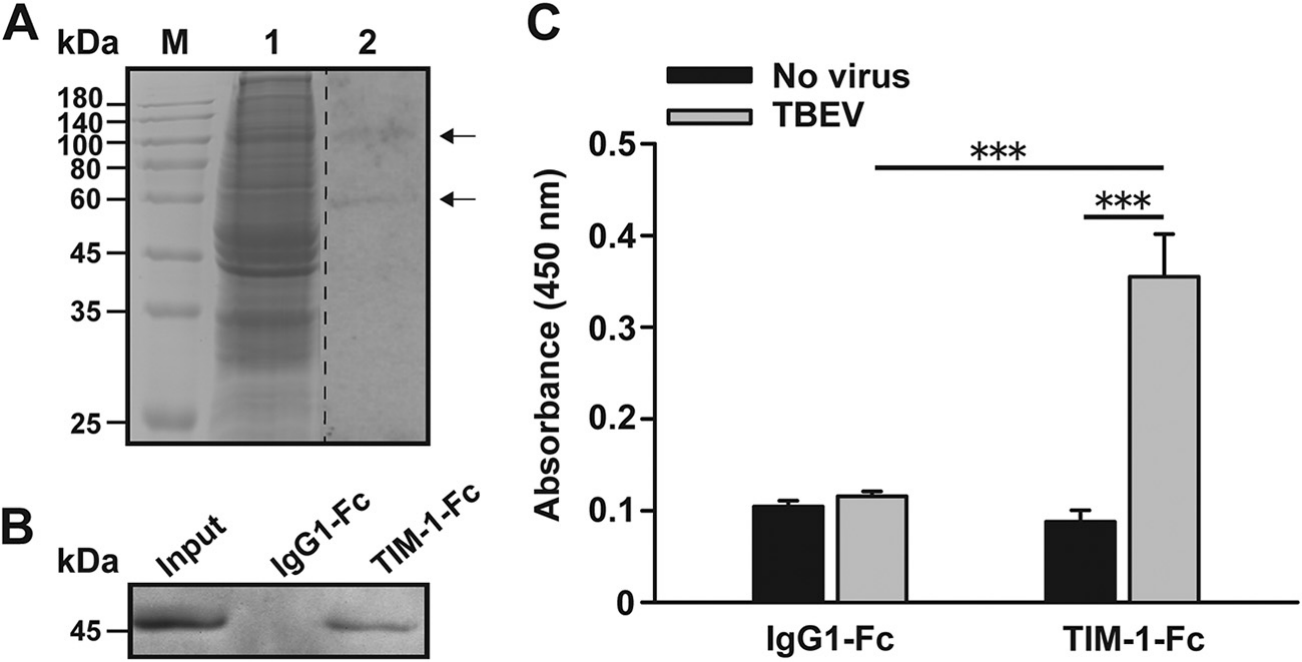
Binding of TBEV to TIM-1. (A) VOPBA was carried out to determine the cell membrane proteins that interact with TBEV. Lanes: M, molecular mass ladder; 1, the Coomassie blue-stained gel used to excise the corresponding bands for LC-MS/MS analysis; 2, TBEV binding to A549 cell membrane proteins visualized by VOPBA. Arrows indicate the bands that were identified in the VOPBA and assessed by LC-MS/MS. Representative images of three independent experiments are shown. (B) Western blot analysis of TBEV preincubated with TIM-1-Fc or IgG-Fc bound to protein G-agarose beads. Pulled-down virus was detected using the anti-E 4G2 MAb. Representative images of three independent experiments are shown. (C) IgG-Fc or TIM-1-Fc was used to coat 96-well plates and incubated with TBEV for 2 h at RT. Bound virus was detected using the 4G2 MAb and HRP-conjugated goat anti-mouse IgG. Data are presented as the mean ± standard deviation of three independent experiments. ***, *P *<* *0.001.

To identify candidate cell membrane proteins that interact with TBEV, we conducted a virus overlay protein binding assay (VOPBA) followed by liquid chromatography-tandem mass spectrometry (LC-MS/MS) analysis. VOPBA using TBEV on membrane proteins extracted from permissive A549 cells (9) revealed several bands. The putative virus-associated proteins were excised from the corresponding gels and analyzed using LC-MS/MS. Of the various hits obtained by MS, TIM-1 (Table 1, shown in bold) with a molecular weight of approximately 100 kDa was found to be a potential candidate (Fig. 1A). TIM-1 is a cell surface glycoprotein that binds to phosphatidylserine (PtdSer) on the surface of apoptotic cells and internalizes apoptotic bodies (10). It serves as the receptor for several viruses through viral apoptotic mimicry (11).

**TABLE 1:** Analysis of putative TBEV-associated proteins by LC-MS/MS

Gene	Description	Accession^*a*^	Score^*b*^	%Cov(95)^*c*^
MTHFD1	C-1-tetrahydrofolate synthase, cytoplasmic	P11586	28.65	14.55000043
IGHG1	Immunoglobulin heavy constant gamma 1	P01857	25.74	29.69999909
KIF5B	Kinesin-1 heavy chain	P33176	16.38	9.76099968
VCL	Vinculin	P18206	15.90	6.877999753
CLTC	Clathrin heavy chain 1	Q00610	13.16	6.328000128
AP2B1	AP-2 complex subunit beta	P63010	6.11	4.695999995
SEC24C	Protein transport protein Sec24C	P53992	6.04	3.931000084
SLC3A2	4F2 cell-surface antigen heavy chain	P08195	5.60	4.602999985
**TIM-1**	**T-cell immunoglobulin mucin receptor 1**	Q96D42	**5.00**	**13.36999983**
DNM1	Dynamin-1	Q05193	4.96	3.934999928
WASHC5	WASH complex subunit 5	Q12768	3.75	1.812000014
HSP90B1	Endoplasmin	P14625	3.44	5.22999987
EMC1	ER membrane protein complex subunit 1	Q8N766	2.51	1.813000068
NCKAP1	Nck-associated protein 1	Q9Y2A7	2.07	0.975199975
ITGAV	Integrin alpha-V	P06756	2.01	1.813000068
VPS18	Vacuolar protein sorting-associated protein 18 homolog	Q9P253	1.32	1.439000014

a Identification number of the protein in the UniProt protein sequence database.

b Unused ProtScore.

c The number of peptides matching the identified protein sequence with confidence greater than 95%.

To verify the association of TBEV with TIM-1, we conducted a pulldown assay with soluble TIM-1-Fc. TBEV particles were incubated with TIM-1-Fc or IgG1-Fc, followed by protein G Sepharose beads. Binding was evaluated by immunoblotting for TBEV envelope (E) protein using the 4G2 MAb. As shown in Fig. 1B, TBEV bound to the TIM-1 construct, but not to the IgG1-Fc negative-control construct. Similar to previous studies (12, 13), soluble TIM-1-Fc immunoprecipitated Ebola virus-like particles (EBOV-VLPs), but not influenza A virus H1N1 (PR8) particles (Fig. S1), confirming the effectiveness of this approach. Additionally, the TBEV-TIM-1 interaction was confirmed by enzyme-linked immunosorbent assay (ELISA). We showed that TBEV reacted with the TIM-1-Fc coating on 96-well plates, whereas it did not react with the IgG1-Fc negative control (Fig. 1C). These data demonstrated an interaction between TBEV and TIM-1.

10.1128/mbio.02860-21.1 FIG S1 Virus particles bind to soluble TIM-1. EBOV-VLPs or influenza A virus H1N1 (PR8) particles were preincubated with soluble TIM-1-Fc or IgG1-Fc, followed by incubation with protein G-agarose beads. Beads were washed to remove unbound virus, and virion binding was evaluated by immunoblotting. Representative images from three independent experiments are shown. Download FIG S1, TIF file, 1.5 MB. Copyright © 2022 Zhang et al. 2022 Zhang et al. https://creativecommons.org/licenses/by/4.0/ This content is distributed under the terms of the Creative Commons Attribution 4.0 International license.

### Ectopic expression of TIM-1 facilitates TBEV infection.

**FIG 2:**
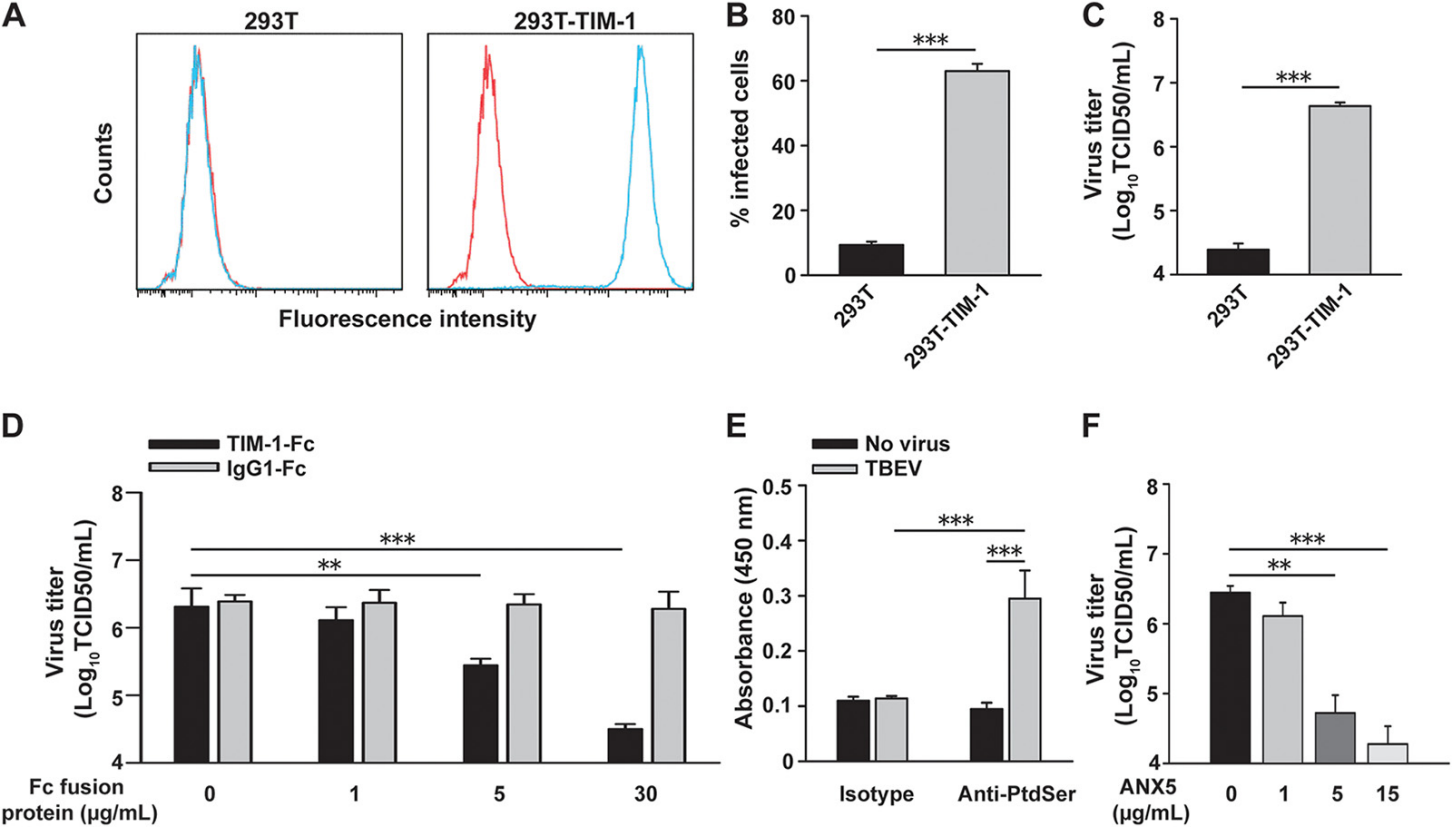
Ectopic expression of TIM-1 facilitates TBEV infection. (A) Surface levels of TIM-1 in 293T and 293T-TIM-1 cells. 293T parental (left panel) and transduced cells (right panel) were stained with anti-TIM-1 (blue) or isotype control (red) antibody. (B) Parental and TIM-1-expressing 293T cells were challenged with TBEV (MOI = 1). The levels of infected cells were assessed 24 h postinfection by flow cytometry using the 4G2 MAb. (C) Supernatants were collected 48 h postinfection, and virus titers were determined by the TCID_50_ assay. (D) TIM-1-Fc treatment inhibited virus infection of 293T-TIM-1 cells. TBEV preincubated with TIM-1-Fc or IgG1-Fc was used to infect 293T-TIM-1 cells (MOI = 1), and virus titers were assessed 48 h postinfection. (E) The wells of 96-well plates were coated with TBEV particles, and PtdSer on virions were detected using the anti-PtdSer MAb and HRP-conjugated goat anti-mouse IgG. (F) ANX5 inhibited TIM-1-mediated enhancement of virus infection of 293T-TIM-1 cells. TBEV preincubated with ANX5 was used to infect 293T-TIM-1 cells (MOI = 1), and virus titers were assessed 48 h postinfection. The results are presented as the means ± standard deviation of three independent measurements. **, *P *<* *0.01; ***, *P *<* *0.001.

Next, we explored the involvement of TIM-1 expression in TBEV infection. Previous studies show that the human embryonic kidney cell line 293T does not express TIM-1 (14). Then parental 293T cells were engineered to express TIM-1 (293T-TIM-1; Fig. 2A). Cells were challenged with TBEV, and infection rates were quantified by flow cytometry. Infection of 293T-TIM-1 cells with TBEV resulted in a remarkable increase in the percentage of virus-infected cells compared with the parental 293T cells (Fig. 2B). Through titration of cell-free supernatants collected from cells challenged with TBEV, we found that 293T-TIM-1 cells supported significantly greater virus replication than the parental cells (*P *<* *0.001; Fig. 2C). We next examined whether soluble TIM-1-Fc can inhibit TBEV infection of 293T-TIM-1 cells. As shown in Fig. 2D, virus infection was inhibited by soluble TIM-1-Fc in a dose-dependent manner. In contrast, control IgG1-Fc did not affect TBEV infection of cells. These data indicate that ectopic TIM-1 expression in poorly permissive cells facilitates TBEV infection.

TIM-1 binds PtdSer, which can be exposed on the surface of viruses (15, 16). To investigate whether the TIM-1 ligand, PtdSer, associates with TBEV, ELISA wells were coated with purified virions and incubated with the specific anti-PtdSer monoclonal antibody (MAb). We found that the anti-PtdSer MAb, but not the isotype control, bound to TBEV-coated ELISA wells (Fig. 2E). To examine whether viral PtdSer correlates with TIM-1-enhanced TBEV infection of 293T-TIM-1 cells, we preincubated TBEV with annexin V (ANX5), a PtdSer-binding protein. ANX5 inhibited TBEV infection of 293T-TIM-1 cells in a dose-dependent manner, suggesting that interaction between PtdSer and TIM-1 could enhance TBEV infection of cells (Fig. 2F). Notably, neither Fc-fused proteins (Fig. S2) nor ANX5 (Fig. S3) treatment led to obvious cytotoxicity. Treatment of cells with Triton X-100 was used as a positive control.

10.1128/mbio.02860-21.2 FIG S2 LDH release from Fc fusion protein-treated cells. 293T-TIM-1 and A549 cells were treated with TIM-1-Fc or IgG1-Fc at the indicated doses for 48 h. Treatment of cells with Triton X-100 for 1 h was used as a positive control. The culture supernatants were centrifuged at low speed and analyzed with the LDH release detection kit. Data are represented as the means ± standard deviations of three independent experiments. Download FIG S2, TIF file, 0.2 MB. Copyright © 2022 Zhang et al. 2022 Zhang et al. https://creativecommons.org/licenses/by/4.0/ This content is distributed under the terms of the Creative Commons Attribution 4.0 International license.

10.1128/mbio.02860-21.3 FIG S3 LDH release of cells resulting from treatment with ANX5. Cells were treated with ANX5 at the indicated doses for 48 h. Treatment of cells with Triton X-100 was used as a positive control. The culture supernatants were collected for LDH release assay. Data are represented as the means ± standard deviations of three independent experiments. Download FIG S3, TIF file, 0.4 MB. Copyright © 2022 Zhang et al. 2022 Zhang et al. https://creativecommons.org/licenses/by/4.0/ This content is distributed under the terms of the Creative Commons Attribution 4.0 International license.

### TIM-1 plays a role in the entry phase of TBEV infection.

**FIG 3:**
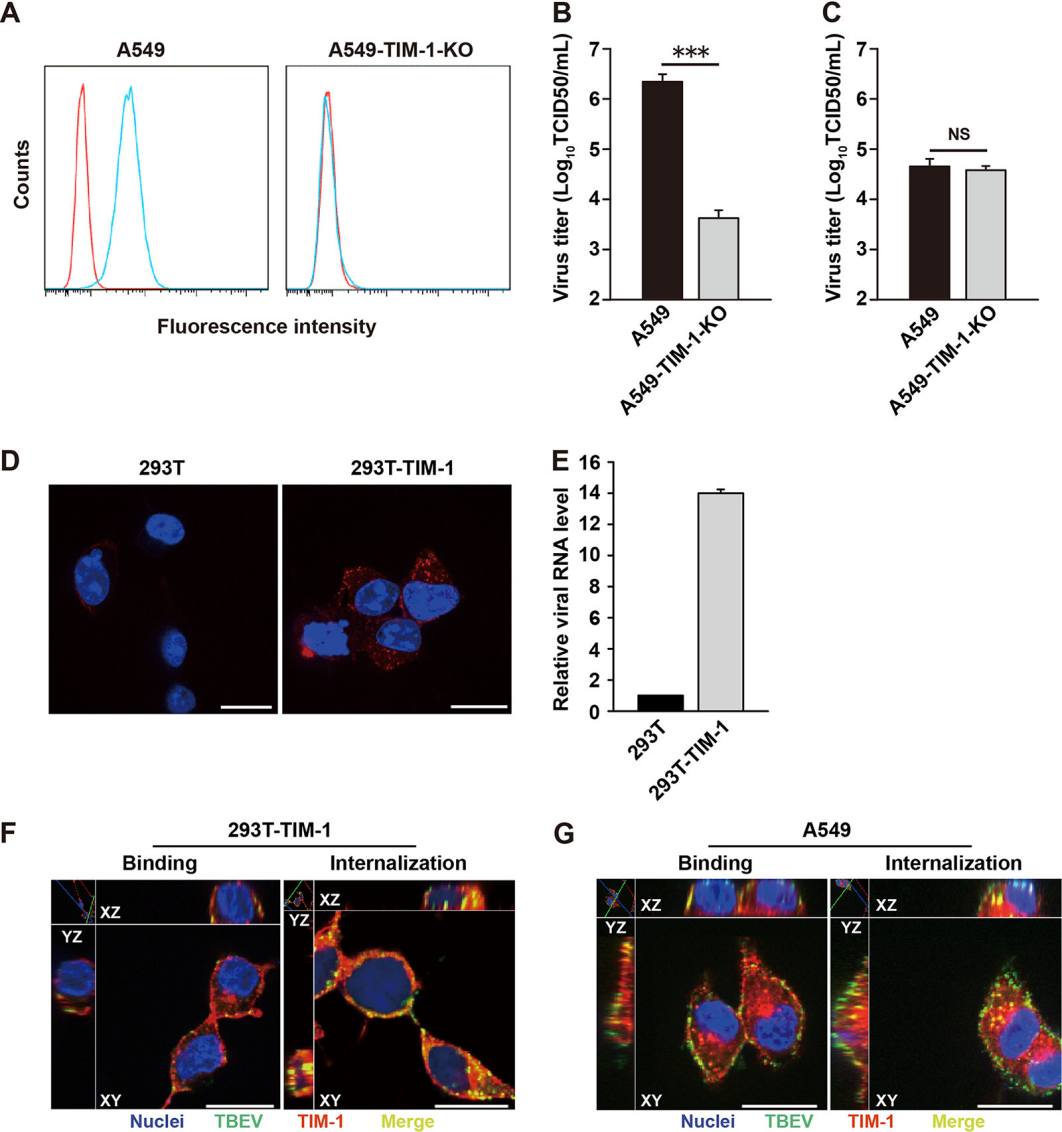
TIM-1 mediates virus entry. (A) TIM-1 cell surface expression in A549 and A549-TIM-1-KO cells. A549 parental (left panel) and A549-TIM-1-KO cells (right panel) were stained with anti-TIM-1 (blue) or isotype control (red) antibody. (B) Parental and TIM-1-KO A549 cells were challenged with TBEV (MOI = 1). Supernatants were collected 48 h postinfection, and virus titers were determined by the TCID_50_ assay. (C) A549 or A549-TIM-1-KO cells were transfected with 5 μg of *in vitro* transcribed viral RNA. Four days posttransfection, supernatant was collected and virus production was quantified by TCID_50_ assay. (D) Parental 293T and 293T-TIM-1 cells were incubated with TBEV (MOI = 5) for 1 h at 4°C, and then at 37°C for 45 min. Cells were stained with Hoechst 33342 (blue, nuclear staining) and 4G2 MAb (red) to determine virus uptake. Representative images of three independent experiments are shown. Scale bar = 20 μm. (E) Total RNA was extracted from infected 293T and 293T-TIM-1 cells, and viral RNA levels were quantified by qRT-PCR with human GAPDH as an endogenous control. Results are expressed as the fold change compared with parental 293T as the calibrator value. Data are presented as the means ± standard deviation of three independent experiments. (F) 293T-TIM-1 and (G) A549 cells were exposed to TBEV (MOI = 10) at 4°C for 1 h, with or without a shift to 37°C for 45 min. Cells were fixed and stained for nucleus (blue), TBEV E protein (green), and TIM-1 (red). Areas of yellow indicate TBEV-TIM-1 colocalization on the cell surface or within the cytoplasm. Samples were observed under a confocal microscope. Representative images of three independent experiments are shown. Scale bar = 20 μm. The results are presented as the means ± standard deviation of three independent measurements. ***, *P *<* *0.001. NS, not significant.

To determine if the requirement for TIM-1 in the TBEV life cycle was at the level of entry, we performed a viral RNA bypass assay. TIM-1 knockout A549 cells (A549-TIM-1-KO) were generated by using clustered regularly interspaced short palindromic repeat (CRISPR)/Cas9-mediated gene editing technology (Fig. 3A). We compared the production of progeny viruses between infection (entry-dependent) and viral RNA transfection (entry-independent) in either A549 or A549-TIM-1-KO cells. As shown in Fig. 3B, TBEV infection was significantly inhibited in A549-TIM-1-KO cells compared to A549 cells (*P *<* *0.001). In contrast, similar levels of infectious virus were detected in either wild-type (WT) or TIM-1 KO cells transfected with viral RNA (Fig. 3C). This demonstrates that the requirement for TIM-1 was largely bypassed by viral RNA transfection, suggesting a role for TIM-1 in entry, but not for a step downstream of entry.

### TIM-1 mediates TBEV entry and is cointernalized with virus particles into cells.

To study the entry of infectious TBEV particles into cells, parental 293T and 293T-TIM-1 cells were incubated with viruses for 1 h at 4°C, followed by 45 min at 37°C to allow endocytosis. Subsequently, these cells were stained for viral E protein using the 4G2 MAb. 293T-TIM-1 cells showed increased intracellular accumulation of viral protein compared with 293T cells (Fig. 3D). Next, total RNA was extracted from virus-infected cells, and viral RNA levels were determined by reverse transcription-quantitative PCR (qRT-PCR). Increased TBEV RNA uptake was observed in 293T-TIM-1 cells compared with the parental 293T cells, suggesting that ectopic TIM-1 enhances virus entry into cells (Fig. 3E).

Then, we performed double immunofluorescence staining for TBEV antigen and TIM-1 to analyze the subcellular localization of the virus and entry factor. Confocal microscopy of 293T-TIM-1 cells incubated with TBEV at 4°C for virus binding without internalization revealed colocalization of TIM-1 and TBEV on the cell surface. When the cells were allowed to internalize the virus at 37°C for 45 min, colocalization of the virus and the TIM-1 was clearly observed within the cytoplasm (Fig. 3F). Similar results were seen in TBEV-infected A549 cells, which express TIM-1 endogenously (17) (Fig. 3G). These data demonstrate that TIM-1 mediates TBEV entry and is cointernalized with virus particles into cells.

### Blocking of endogenous TIM-1 inhibits TBEV infection.

**FIG 4:**
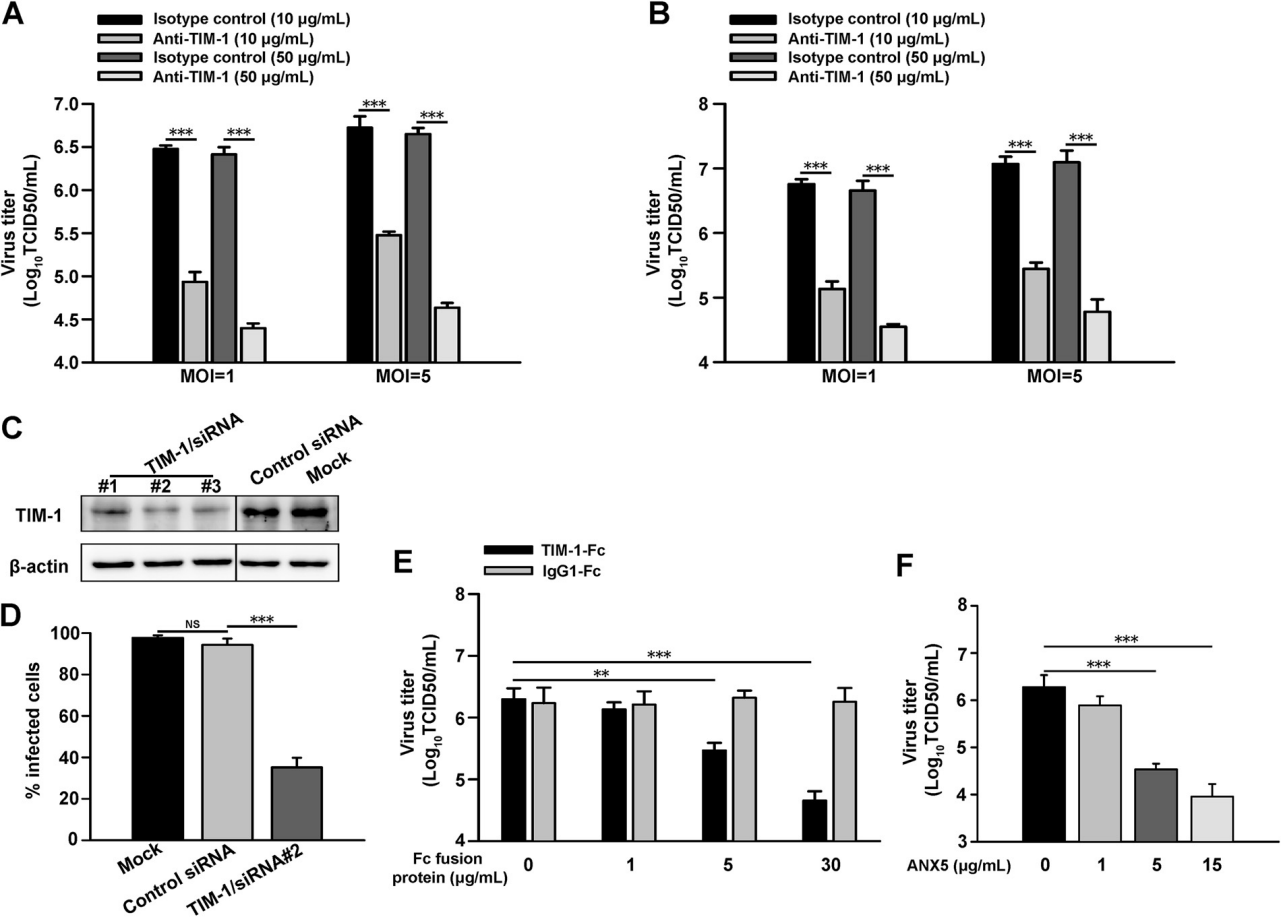
Blocking of endogenous TIM-1 inhibits TBEV infection. (A) A549 and (B) Vero cells were infected with TBEV (MOI = 1 or 5) in the presence of anti-TIM-1 or isotype control MAbs. Supernatants were collected 48 h postinfection, and virus titers were determined by the TCID_50_ assay. The results are presented as the means ± standard deviation of three independent measurements. (C) TIM-1/siRNA (#1, #2, and #3) or nontargeting control siRNA were transfected into A549 cells via Lipofectamine RNAiMax. Cell lysates were analyzed by Western blotting using the anti-TIM-1 antibody and anti-β-actin antibody. Representative images of three independent experiments are shown. (D) A549 cells were transfected with TIM-1/siRNA (#2) or nontargeting control siRNA, followed by infection with TBEV (MOI = 5) for 24 h. The levels of infected cells were assessed by flow cytometry using 4G2 MAb. (E) TIM-1-Fc treatment inhibited virus infection of A549 cells (MOI = 1). (F) ANX5 inhibited virus infection of A549 cells (MOI = 1). The results are presented as the means ± standard deviation of three independent measurements. **, *P *<* *0.01; ***, *P *<* *0.001. NS, not significant.

We then analyzed the effects of neutralizing anti-TIM-1 antibodies (Abs) on TBEV infection in A549 and Vero cells, which endogenously express TIM-1. Treatment with Abs had no effect on cell viability (Fig. S4). Anti-TIM-1 Abs significantly inhibited infection of A549 cells, whereas control Abs had little effect on virus infection (Fig. 4A). Inhibition of TBEV infection by anti-TIM-1 Abs was also observed in Vero cells (Fig. 4B).

10.1128/mbio.02860-21.4 FIG S4 LDH release from Ab-treated cells. Cells were treated with anti-TIM-1 MAbs or isotype control Abs at the indicated doses for 48 h. Treatment of cells with Triton X-100 was used as a positive control. The culture supernatants were collected for LDH release assay. Data are represented as the means ± standard deviations of three independent experiments. Download FIG S4, TIF file, 1.8 MB. Copyright © 2022 Zhang et al. 2022 Zhang et al. https://creativecommons.org/licenses/by/4.0/ This content is distributed under the terms of the Creative Commons Attribution 4.0 International license.

Next, TIM-1 was silenced by RNA interference in A549 cells. Then, 48 h after TIM-1/small interfering RNA (siRNA) transfection, TIM-1 expression was decreased compared with nontargeting control siRNA (Fig. 4C). Viral infection was verified using TIM-1/siRNA#2 because it had the best knockdown efficiency. As shown in Fig. 4D, TIM-1/siRNA knockdown markedly inhibited TBEV infection (*P *<* *0.001), indicating that TIM-1 has an important role in TBEV infection. Knockdown did not affect cell viability (Fig. S5). Moreover, as for TIM-1-expressing 293T cells, TIM-1-Fc (Fig. 4E) or ANX5 (Fig. 4F) treatment significantly inhibited TBEV infection of A549 cells.

10.1128/mbio.02860-21.5 FIG S5 Measurement of LDH release after siRNA transfection. A549 cells were transiently transfected with siRNAs for TIM-1 or a negative-control siRNA. The culture supernatants were collected 72 h posttransfection, and LDH release was measured. Treatment of cells with Triton X-100 was used as a positive control. Data are represented as the means ± standard deviations of three independent experiments. Download FIG S5, TIF file, 0.1 MB. Copyright © 2022 Zhang et al. 2022 Zhang et al. https://creativecommons.org/licenses/by/4.0/ This content is distributed under the terms of the Creative Commons Attribution 4.0 International license.

### TIM-1 promotes TBEV infection in primary cells.

**FIG 5:**
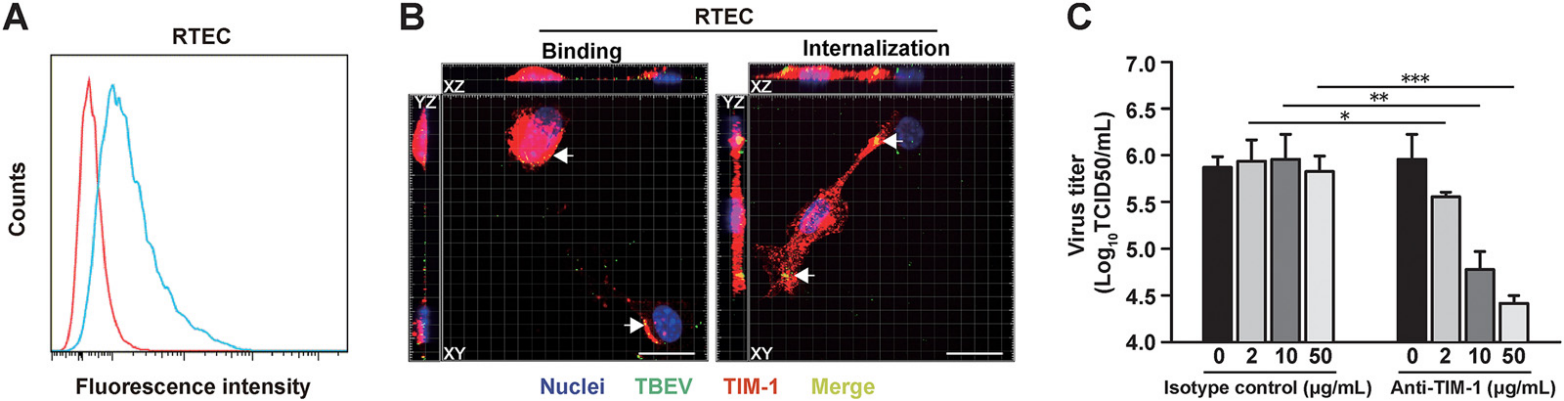
TIM-1 promotes TBEV infection in primary cells. (A) Surface expression of TIM-1 on RTEC was quantified by flow cytometry. A representative histogram shows the measured fluorescence of cells incubated with anti-TIM-1 (blue) or isotype control (red) antibody. (B) RTEC were exposed to TBEV (MOI = 5) at 4°C for 1 h, with or without a shift to 37°C for 45 min. Cells were fixed and stained for nucleus (blue), TBEV E protein (green), and TIM-1 (red). Yellow and arrows indicate TBEV-TIM-1 colocalization on the cell surface or within the cytoplasm. Samples were observed under a confocal microscope. Representative images of three independent experiments are shown. Scale bar = 20 μm. (C) TBEV infection of RTEC is inhibited by antibodies to TIM-1 in a dose-dependent manner. Supernatants were collected 48 h postinfection, and virus titers were determined by the TCID_50_ assay. The results are presented as the means ± standard deviation of three independent measurements. *, *P *<* *0.05; **, *P *<* *0.01; ***, *P *<* *0.001.

To prove that endogenous TIM-1 is important for entry of TBEV in primary cells, mouse primary renal tubular epithelial cells (RTEC) were utilized as a relevant cell type. It was shown that TBEV viral RNA could be detected in kidney and urine samples of humans and other natural hosts even if viremia was not detected, which would suggest infection of renal parenchymal cells (18–20). TIM-1 expression has been demonstrated in epithelial cells of kidney origin and is upregulated in both mouse and human kidneys after injury (14). We performed flow cytometry analyses to measure the TIM-1 expression level. TIM-1 was detected on the cell surface of RTEC as judged by antibody reactivity (Fig. 5A). Colocalization of TIM-1 and TBEV was detected both on the cell surface and within the cytoplasm of RTEC (Fig. 5B). In blocking experiments, infection of RTEC was inhibited by the TIM-1-specific antibodies in a dose-dependent manner (Fig. 5C). Treatment with Abs did not cause obvious cytotoxicity (Fig. S6). These results indicate that TIM-1 promotes TBEV infection in permissive cells naturally expressing the molecule.

10.1128/mbio.02860-21.6 FIG S6 LDH release from Ab-treated RTEC. RTEC were treated with anti-TIM-1 MAbs or isotype control Abs at the indicated doses for 48 h. Treatment of cells with Triton X-100 was used as a positive control. The culture supernatants were collected for LDH release assay. Data are represented as the means ± standard deviations of three independent experiments. Download FIG S6, TIF file, 0.2 MB. Copyright © 2022 Zhang et al. 2022 Zhang et al. https://creativecommons.org/licenses/by/4.0/ This content is distributed under the terms of the Creative Commons Attribution 4.0 International license.

### TIM-1 deficiency attenuates TBEV infection and pathogenesis in mice with a defective interferon (IFN) system.

**FIG 6:**
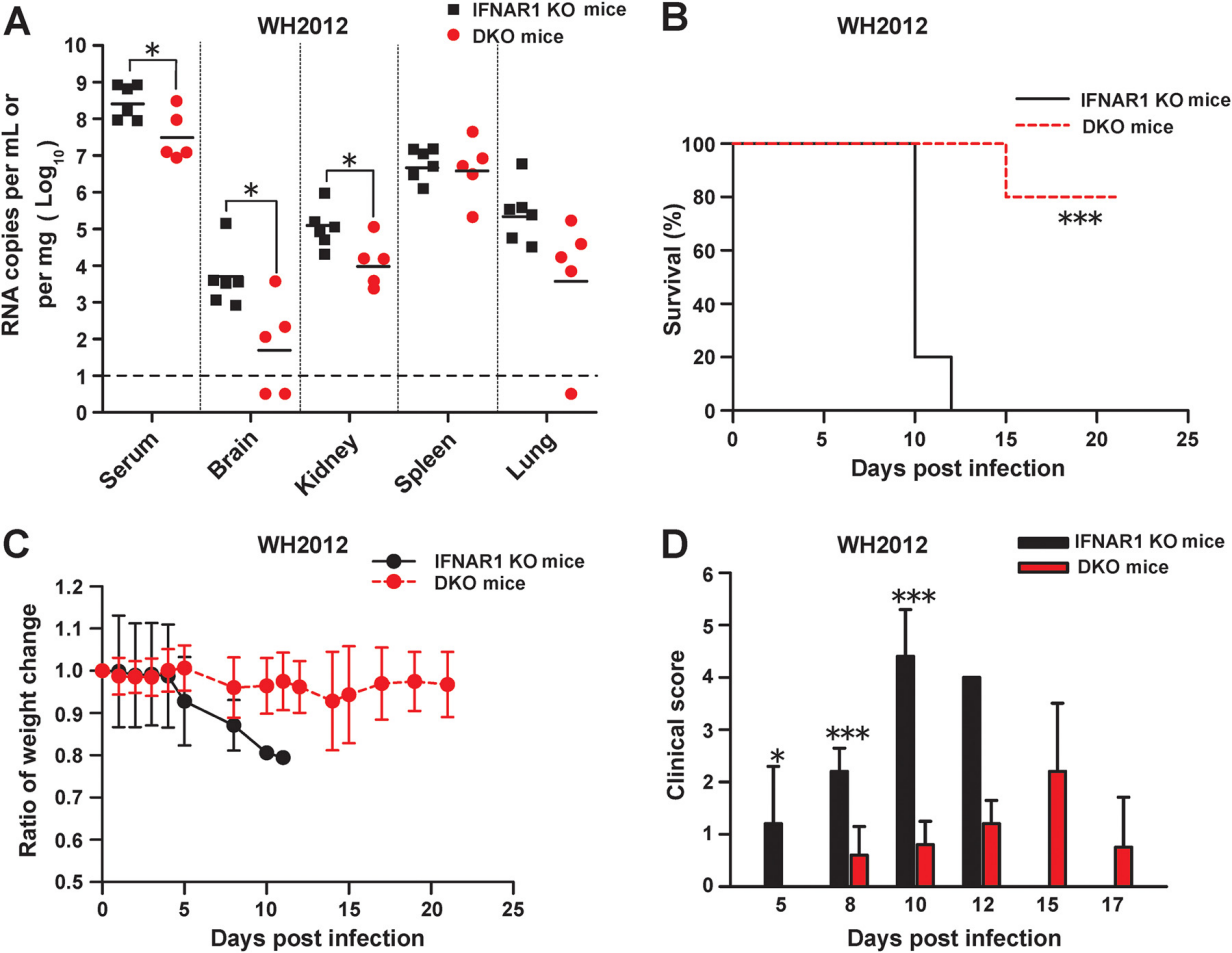
TIM-1 deficiency attenuates TBEV infection and pathogenesis in mice with a defective IFN system. DKO or IFNAR1 KO mice were infected with TBEV WH2012 (25 TCID_50_) via a footpad injection. (A) Viral RNA levels were determined by qRT-PCR 8 days postinjection in the indicated tissues. The numbers of viral RNA copies per mg tissue or per mL serum are reported. Each line segment represents the mean of samples (points) pooled from two independent experiments (*n* = 5 to 6). The horizontal dashed line marks the limit of detection (LOD) of the assay. All negative samples are plotted as the half value of the LOD. (B) Survival was assessed following infection, and significance for the survival curve was determined by the Kaplan-Meier log rank test (*n* = 5). (C) The body weight change ratio (compared to day 0) in TBEV WH2012-infected DKO and IFNAR1 KO mice was calculated for each recording day. Error bars represent standard deviations. (D) Animals were monitored for clinical manifestations. Signs of illness were scored as follows: 0, no symptoms; 1, ruffled fur or hunched posture; 2, asthenia or paresis; 3, lethargy, tremor, or paralysis; 4, moribund or euthanized; and 5, death. Error bars represent standard deviations. *, *P *<* *0.05; ***, *P *<* *0.001.

The contribution of TIM-1 to TBEV infection *in vivo* was further tested. We first used mice deficient in type I interferon receptor (IFNAR1 KO mice), as these are highly susceptible to flavivirus infection and disease progression (21, 22). To explore the impact of TIM-1 on TBEV pathogenesis, homozygous TIM-1^−/−^ IFNAR1^−/−^ double KO (DKO) mice were generated and used in this study. DKO and IFNAR1 KO mice were challenged with TBEV strain WH2012 via a footpad injection to mimic the primary route of virus transmission. We measured viral RNA loads in serum and infected organs of both DKO and IFNAR1 KO mice. On day 8 postchallenge, the levels of viral RNA in the serum (*P *<* *0.05), brain (*P *<* *0.05), and kidneys (*P *<* *0.05) of DKO mice were significantly reduced compared with those of IFNAR1 KO mice (Fig. 6A). One hundred percent of TIM-1-sufficient IFNAR1 KO mice succumbed to virus by day 12 postinfection. In contrast, DKO mice challenged with TBEV had markedly reduced mortality (Fig. 6B). DKO mice (19.8 ± 2.7 days) exhibited a significantly longer mean survival time in comparison with IFNAR1 KO mice (10.4 ± 0.9 days) (*P *<* *0.001). Weight loss was observed in both groups; however, the surviving DKO mice almost completely recovered by day 21 postinfection (Fig. 6C). All IFNAR1 KO mice showed severe signs of illness, such as paresis, hind limb paralysis, or tremor. In comparison, only 20% of DKO mice infected with TBEV demonstrated severe neurological symptoms (Fig. 6D).

### TIM-1 deficiency reduces viral tissue burden and pathogenicity in immunocompetent mice.

**FIG 7:**
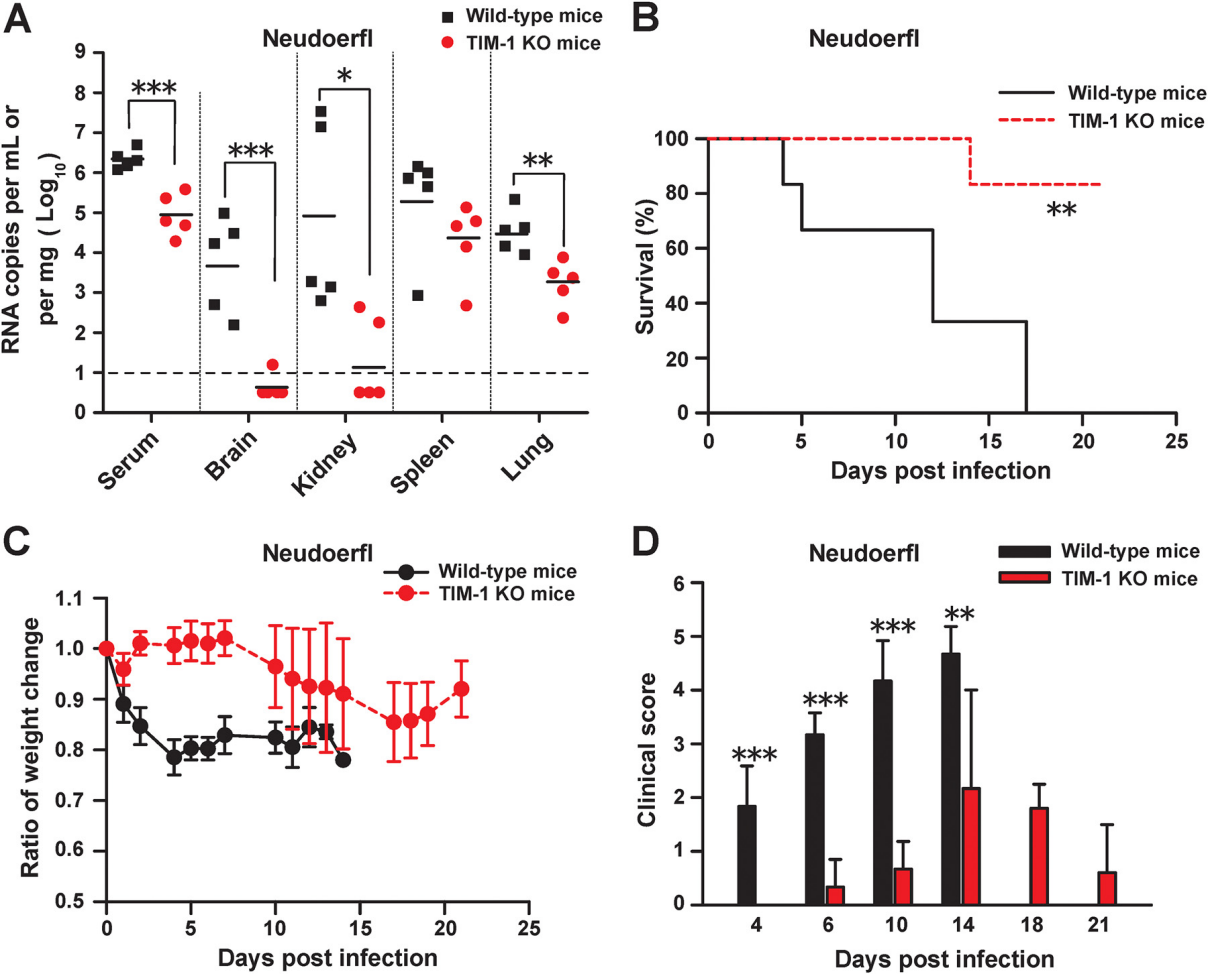
TIM-1 deficiency reduces viral tissue burden and pathogenicity in immunocompetent mice. WT or TIM-1 KO mice were infected with TBEV Neudoerfl (1 × 10^5^ TCID_50_) via a footpad injection. (A) Viral RNA levels in the indicated tissues were measured on day 5 postinfection. Each line segment represents the mean of samples (points) pooled from two independent experiments (*n* = 5). (B) Survival was assessed following infection, and the significance for survival curve was determined by the Kaplan-Meier log rank test (*n* = 6). (C) The body weight change ratio in TBEV Neudoerfl-infected WT and TIM-1 KO mice was calculated for each recording day. Error bars represent standard deviations. (D) Animals were monitored for clinical manifestations. Signs of illness were scored as follows: 0, no symptoms; 1, ruffled fur or hunched posture; 2, asthenia or paresis; 3, lethargy, tremor, or paralysis; 4, moribund or euthanized; and 5, death. Error bars represent standard deviations. *, *P *<* *0.05; **, *P *<* *0.01; ***, *P *<* *0.001.

In further support, we assessed the impact of TIM-1 on TBEV infection in an immunocompetent mouse model. WT C57BL/6 mice and TIM-1 knockout (TIM-1 KO) littermates were infected with TBEV by the footpad route. Though no deaths were observed in either TIM-1 KO or WT mice during the 21 days after infection of strain WH2012, we observed significantly lower viral RNA loads in the serum (*P *<* *0.001), brain (*P *<* *0.05), and lungs (*P *<* *0.05) of TIM-1 KO mice compared with the WT group (Fig. S7). To validate the contribution of TIM-1 to TBEV infection *in vivo*, strain Neudoerfl, which displayed virulence in WT mice, was used for the following study. We found that the viral loads in the serum (*P *<* *0.001), brain (*P *<* *0.001), kidneys (*P *<* *0.05), and lungs (*P *<* *0.01) of WT mice were significantly higher than in those of TIM-1 KO mice at day 5 postinfection (Fig. 7A). WT mice were found to be highly susceptible to infection, displaying 100% mortality, compared to 16.7% mortality in TIM-1 KO mice (Fig. 7B). Obvious weight loss was observed in WT mice at the early stage of infection. TIM-1 KO mice lost weight much more slowly, and their body weight bounced back by day 21 postinfection (Fig. 7C). WT mice infected with strain Neudoerfl developed severe neurological signs, while the TIM-1 KO mice demonstrated moderate clinical signs (Fig. 7D). Taken together, our findings provide evidence that TIM-1 is associated with viral burden and pathogenesis for TBEV *in vivo*.

10.1128/mbio.02860-21.7 FIG S7 TIM-1 KO or wild-type mice were infected with TBEV WH2012 (1 × 10^5^ TCID_50_) via a footpad injection. Viral RNA levels were determined by qRT-PCR 6 days postinjection in the indicated tissues. The numbers of viral RNA copies per mg tissue or per mL serum are reported. Each line segment represents the mean of samples (points) pooled from two independent experiments (*n* = 5). The horizontal dashed line marks the limit of detection (LOD) of the assay. All negative samples are plotted as the half value of the LOD. *, *P *<* *0.05; ***, *P *<* *0.001. Download FIG S7, TIF file, 0.10 MB. Copyright © 2022 Zhang et al. 2022 Zhang et al. https://creativecommons.org/licenses/by/4.0/ This content is distributed under the terms of the Creative Commons Attribution 4.0 International license.

## DISCUSSION

To date, knowledge regarding TBEV entry into the host cell remains limited, and the host determinants required for TBEV entry have yet to be identified and characterized. Here, we found that TIM-1 is a functional entry factor for TBEV infection *in vitro* and *in vivo*.

In this study, we provide several lines of evidence establishing TIM-1 as a key host factor for TBEV entry. Ectopic expression of TIM-1 significantly increased TBEV infection of poorly permissive cells. SiRNA-mediated knockdown of TIM-1 expression or CRISPR/Cas9-mediated knockout of the *TIM-1* gene markedly decreased TBEV entry into permissive cell lines. Therefore, the presence of TIM-1 correlated directly with susceptibility of cells to TBEV infection. Antibodies directed against TIM-1 or soluble TIM-Fc were capable of potently inhibiting TBEV infection. This indicates that blocking viral access to TIM-1 on the cell surface substantially limits TBEV infection, and the role of TIM-1 occurs at the interface of the cell. Moreover, the ability of anti-TIM-1 Abs or PtdSer ligand to inhibit TBEV infection of cells expressing TIM-1 may provide an effective antiviral therapy (16, 23). Pharmacological inhibition of such cellular components rather than viral proteins may provide useful antiviral agents and reduce the rate of development of antiviral resistance to therapeutic agents (14, 24).

The viral burden was significantly decreased in the organs of TIM-1-deficient mice. Considering the endogenous expression of TIM-1 in keratinocytes (25), lymphocytes (26), human brain tissue (27), kidney cells (10, 28), and lung-derived cells (29), the TBEV-TIM-1 interaction may be relevant to viral tissue tropism. We proved that endogenous TIM-1 was important for entry of TBEV in mouse primary RTEC of kidney origin, suggesting the involvement of TIM-1-mediated TBEV entry under physiological conditions. Thus, the TBEV/TIM-1 interaction may be relevant to *in vivo* TBEV infection and tissue tropism. Future work needs to further clarify the impact of TIM-1 expression on TBEV infection of specific cell types of the susceptible organs.

We have shown that TIM-1 deficiency decreased mortality and morbidity in both IFNAR1 KO and immunocompetent mice when challenged with TBEV. The viral entry mediated by TIM-1 may be one of the determinants of TBEV pathogenesis. Other consequences resulting from virus-TIM-1 interaction, such as induction of a massive release of inflammatory mediators, may also contribute to the pathogenesis in mice (30, 31). Lower levels of viral loads and milder pathogenesis were observed in mice with a defective TIM-1 after challenge with either strain WH2012 or Neudoerfl, suggesting that the role of TIM-1 in mediating virus infection was not strain- or subtype-specific. Overall, the enhanced survival and reduced viral burden in the TIM-1-deficient mice suggest that TIM-1 serves as a host factor that mediates TBEV infection *in vivo*. It is noteworthy that treatment with anti-TIM-1 Abs did not completely inhibit TBEV infection, and virus infection levels were not completely abolished in A549-TIM-1-KO cells. Besides, comparable levels of viral burden could be detected in infected spleens of both TIM-1-deficient and -sufficient mice, indicating that possible alternative entry pathways were present. Hence, TIM-1-mediated entry may be one of the main mechanisms, but not the exclusive means, for entry of TBEV.

In summary, the present findings of this study indicate that TIM-1 serves as a host factor for cellular entry of TBEV. Characterization of the TBEV-TIM-1 interaction will shed light on the early events of virus infection and facilitate the development of strategies to intervene and prevent efficient viral entry.

## MATERIALS AND METHODS

### Ethics statement.

This study was conducted in accordance with the guidance of the Institutional Animal Care and Use Committee of Wuhan Institute of Virology, Chinese Academy of Sciences. All surgeries were performed under general anesthesia, and all efforts were made to minimize the number of animals used and reduce their suffering.

### Cell cultures and virus preparation.

A549 (ATCC CCL-185), Vero (ATCC CCL-81), and 293T (ATCC CCL-3216) cells were maintained in Dulbecco modified Eagle medium (DMEM) supplemented with 10% fetal bovine serum (FBS), 2 mM l-glutamine, 100 U/mL penicillin, and 100 mg/mL streptomycin (Life Technologies). Mouse primary renal tubular epithelial cells (RTEC) were obtained from Procell Life Science & Technology (Procell) and cultured in epithelial cell medium supplemented with epithelial cell growth supplement (Procell) and penicillin/streptomycin solution. The TBEV Far-Eastern subtype strain WH2012 was isolated and propagated as described previously (32). TBEV European subtype prototypic strain Neudoerfl was derived from a full-length cDNA clone, pTNd/c, which was generously provided by F. X. Heinz (Medical University of Vienna, Austria). Neudoerfl stocks were prepared and amplified as previously reported (33). Virus titer was determined by the 50% tissue culture infective dose (TCID_50_) assay on Vero cells. Ebola virus-like particles (EBOV-VLPs), with the matrix protein VP40 fused with enhanced green fluorescent protein (EGFP), were prepared as described (34, 35). Influenza A virus H1N1 (PR8) was grown and titrated as previously reported (36).

### VOPBA and MS analysis.

To identify putative molecules on the cell plasma membrane involved in virus binding, VOPBA was carried out as described previously (37, 38). Briefly, the membrane-associated proteins of A549 cells were extracted using a ProteinExt mammalian membrane protein extraction kit (TransGen Biotech). A549 cells have been found to support robust growth of TBEV (9), as well as other tick-borne flaviviruses (39). Cell membrane proteins were separated on a 10% SDS-PAGE gel and then transferred to a polyvinylidene difluoride (PVDF) membrane (Millipore). The membrane was blocked overnight at 4°C with 5% skim milk and 0.5% bovine serum albumin (BSA) in Tris-buffered saline (TBS) and then incubated with purified TBEV in 1% skim milk in TBS overnight at 4°C. After three rinses with TBS, the membrane was reacted with the anti-flavivirus group-specific MAb 4G2 (Millipore), followed by horseradish peroxidase (HRP)-conjugated rabbit anti-mouse IgG antibodies (Sigma-Aldrich). The VOPBA membrane and the SDS-PAGE gel were aligned, and the area in the gel corresponding to the positive band in the VOPBA was dissected and sent to Wuhan Spec-Ally Biotech Co. Ltd. for LC-MS/MS analysis.

### Establishment of 293T cells stably overexpressing TIM-1.

Human TIM-1 cDNA, kindly provided by Shan-Lu Liu (Ohio State University) (40), was subcloned into pCDH-Puro vector (System Biosciences) to generate pCDH-TIM-1. Recombinant lentiviruses containing the transgene were produced by triple transfection of pCDH-TIM-1, pCMV-dR8.91, and pCMV-VSVG (Addgene) in 293T cells. Two days after transfection, lentivirus-containing supernatants were harvested and filtered through a 0.45-μm membrane. TIM-1-null 293T cells were then exposed to fresh lentivirus-laden supernatants in the presence of Polybrene (6 μg·mL^−1^), and transduced cell populations were selected with puromycin (2 μg·mL^−1^). 293T-TIM-1 cells ectopically expressing TIM-1 were generated, and transgene expression was confirmed by flow cytometry analysis.

### CRISPR/Cas9-mediated knockout of TIM-1 expression.

The 293T cells were transfected with a mixture of LentiCRISPRV2 plasmid encoding TIM-1-specific guide RNA (gRNA) or the gRNA control (25), pCMV-dR8.91, and pCMV-VSVG. The recombinant lentivirus expressing CRISPR/Cas9 and specific single guide RNAs (sgRNAs) were collected and used for transduction of A549 cells. Transduced cells were selected by puromycin treatment (3 μg·mL^−1^), and surviving clones were sorted for TIM-1 negative expression (A549-TIM-1-KO) by flow cytometry analysis.

### TBEV RNA transfection.

The full-length DNA (pTNd/c) was linearized and purified as previously described (41). *In vitro* transcription was carried out to generate viral genomic RNA using a MEGAscript kit, in the presence of a cap analog (Ambion). After extraction via TRIzol LS reagent (Invitrogen), the RNA was quantified by spectrophotometry and stored at −80°C until use. A549 and A549-TIM-1-KO cells were electroporated with viral genomic RNA, and virus titer was determined by the TCID_50_ assay at the time points indicated.

### Virus pulldown.

Virus particles were incubated overnight at 4°C with 2 μg of Fc-chimera protein TIM-1-Fc (AdipoGen) or IgG1-Fc (Sino Biological) in TBS containing 10 mM CaCl_2_. BSA-saturated protein G Sepharose beads (GE Healthcare) were added and incubated for 4 h at 4°C. Beads were washed with TBS containing 10 mM CaCl_2_ and 0.05% Tween, and bound material was resolved in Laemmli buffer under nonreducing conditions, followed by loading onto 10% SDS-PAGE gels and electroblotting onto PVDF membranes. PVDF-bound virus particles were detected with the anti-flavivirus group-specific MAb 4G2, anti-EGFP MAb (Abcam) recognizing fused VP40 of EBOV-VLPs, or anti-matrix protein 1 (M1) of influenza A virus H1N1 (PR8) MAb (Sino Biological), followed by incubation with HRP-conjugated rabbit anti-mouse IgG antibodies (Sigma-Aldrich).

### ELISA binding assay.

Wells (96-well plates) were first coated with Fc-fused proteins TIM-1-Fc or IgG1-Fc (400 ng/well) in TBS supplemented with 10 mM CaCl_2_ overnight at 4°C. Wells were washed with PBS with Tween 20 (PBST) and blocked for 2 h at 37°C with TBS containing 10 mM CaCl_2_ and 2% BSA. TBEV particles (5 × 10^5^ TCID_50_) were added and incubated for 2 h at room temperature (RT), followed by rinses with PBST. The 4G2 MAb was added to the wells and incubated for 2 h at RT. After washes with PBST, the plate was incubated with HRP-conjugated goat anti-mouse IgG antibodies (ProteinTech). After five washes with PBST, the plate was incubated with 3,3′,5,5′-tetramethyl benzidine (TMB; Boster Biotechnology) for 30 min at RT in the dark. The reaction was stopped by addition of 4N H_2_SO_4_. The intensity of yellow color developed by conversion of the substrate was measured at 450 nm with a microplate spectrophotometer reader (BioTek). PtdSer was detected on bound TBEV virions (2 × 10^6^ TCID_50_) using anti-PtdSer 1H6 MAb (Millipore) and HRP-conjugated goat anti-mouse MAb.

### Flow cytometry assays.

TIM-1 surface expression was detected by flow cytometry as described previously (42). Briefly, dispersed cells were incubated with anti-human TIM-1, anti-mouse TIM-1, or the corresponding isotype control MAb (BioLegend) for 30 min at 4°C. Cells were washed and pelleted by centrifugation before resuspension in solution containing Alexa 594-conjugated goat anti-mouse or goat anti-rat IgG antibodies (Abcam). Following incubation for 30 min at 4°C in the dark, the cells were washed twice before flow cytometry analysis (fluorescence-activated cell sorter [FACS] LSRFortessa; BD Biosciences). Intracellular viral antigens were stained with the anti-E 4G2 MAb. Infected cells were fixed and permeabilized by prechilled methanol. Then, cells were incubated with primary 4G2 MAb for 1 h at 4°C, followed by two washes. Cells were then incubated with the secondary Alexa 594-conjugated goat anti-mouse Ab for 30 min at 4°C. Cells were washed twice prior to flow cytometry analysis.

### Infection inhibition assay.

TBEV incubated with the indicated doses of ANX5 (Abcam) or TIM-1-Fc in serum-free medium was used to infect TIM-1-expressing cells, including 293T-TIM-1 and A549, at a multiplicity of infection (MOI) of 1. Virus titers were assessed 48 h later by the TCID_50_ assay. The effect of ANX5 and TIM-1-Fc on cellular viability was measured by the lactate dehydrogenase (LDH) assay as described previously (43, 44). Treatment with 10 μL of Triton X-100/well served as a positive control for cytotoxicity.

### Indirect immunofluorescence and confocal microscopy.

Cells were cultured on 35-mm-diameter plastic culture dishes (Nunc) and incubated with TBEV for 1 h at 4°C, with or without a temperature shift to 37°C for 45 min. Cells were fixed with chilled methanol and incubated with primary goat anti-TIM-1 polyclonal Ab (R&D Systems) and mouse anti-E 4G2 MAb. After washing with PBS, cells were incubated with the secondary antibodies, Alexa Fluor 488-conjugated donkey anti-mouse IgG and Alexa Fluor 647-conjugated donkey anti-goat IgG (Abcam). The cell nuclei were counterstained with Hoechst 33342. Cells were visualized under a PerkinElmer UltraVIEW VoX live-cell imaging system (PerkinElmer) or a Dragonfly spinning confocal system (Andor Technology).

### TIM-1 MAb inhibition of infection assay.

Cells were preincubated with media containing the indicated quantities of anti-human TIM-1, anti-mouse TIM-1, or the corresponding isotype control MAb (BioLegend) for 30 min prior to infection. Cells were then infected with TBEV at an MOI of 1 or 5. The presence of antibodies in the cell culture medium was maintained throughout the experiment. Supernatants were collected 48 h later, and virus titers were determined by the TCID_50_ assay.

### RNA interference knockdown.

A549 cells were transiently transfected using the Lipofectamine RNAiMax protocol (Life Technologies) with 10 nM siRNAs, according to the manufacturer’s instructions. After 48 h, cells were harvested for Western blot analysis or infected with TBEV at an MOI of 5, and infected cell percentages were quantified 24 h postinfection by flow cytometry. Pools of three siRNAs for TIM-1 and one negative-control siRNA were purchased from Cohesion Biosciences. The effect of siRNAs on cell viability was measured by the LDH assay.

### Quantitation of viral RNA uptake.

Parental 293T and TIM-1-expressing 293T-TIM-1 cells were incubated with TBEV for 1 h at 4°C, followed by 37°C for 45 min. Cells were then treated with proteinase K (Beyotime; final concentration, 1 mg/mL) for 45 min at 4°C to remove noninternalized virus particles. Total RNA was extracted from infected cells using an Omega HP total RNA isolation kit (Omega Bio-Tek, Inc.). The viral RNA level was determined by real-time quantitative PCR (qRT-PCR) with human GAPDH (glyceraldehyde-3-phosphate dehydrogenase) as the endogenous control. QRT-PCR was carried out using a HiScript II one-step qRT-PCR SYBR green kit (Vazyme) on a Bio-Rad CFX96 real-time PCR system (Bio-Rad Laboratories, Inc.). The following primers were used: TBEV-F (genome position 7656 to 7675 relative to TBEV strain WH2012, GenBank accession no. KJ755186), 5′-TAGGCGTGGTGGTTCTGAGG-3″; TBEV-R (genome position 7846 to 7827), 5′-GTTTAGCCGTGCCCCGTGAC-3″; GAPDH-F, 5′-GAAGGTGAAGGTCGGAGTC-3″; GAPDH-R, 5′-GAAGATGGTGATGGGATTTC-3″ (45). Results are expressed as the fold difference using expression in 293T infected cells as the calibrator value.

### Mice and virus infection.

Heterozygous C57BL/6J-TIM-1^+/–^ mice (B6/JNju-*Havcr1^em1Cd19184^*/Nju) were generated at Nanjing Biomedical Research Institute of Nanjing University (NBRI) using CRISPR/Cas9 targeting the genomic region between exon 2 and exon 5. C57BL/6J-IFNAR1^−/−^ (B6.129S2-*Ifnar1^tm1Agt^*/Mmjax, IFNAR1 KO) mice were kindly provided by Xinwen Chen (Wuhan Institute of Virology, Chinese Academy of Sciences). C57BL/6J-TIM-1^−/−^ mice (TIM-1 KO) were produced by crossing parental C57BL/6J-TIM-1^+/–^ mice. TIM-1^−/−^ IFNAR1^−/−^ double KO (DKO) mice were generated by crossing TIM-1 KO and IFNAR1 KO mice. Mice were interbred, and genomic DNA from progeny was genotyped by PCR. The TIM-1 genotyping primers included the following: shared forward primer, 5′-TGTACGAGCTGTCTGCTGTTACTAG-3′; KO reverse primer, 5′-TCTGGCAGCTCAAACCCAAG-3′; WT reverse primer, 5′-TATGCCTGCACTCTCAGGCCA-3′. PCR amplification was carried out by 35 cycles of 15 s at 94°C, 15 s at 65°C, and 1 min at 72°C. The primers and protocol for IFNAR1^−/−^ screening have been previously described (46). All expected genotypes were generated in normal Mendelian ratios. Homozygous progeny of TIM-1 KO and WT littermates at 6 to 8 weeks of age, or homozygous progeny of DKO and IFNAR1 KO at 10 to 12 weeks of age were infected via footpad injection using concentrations of virus noted in the figure legends. All inoculums were administered in the left-rear footpad. Animals were weighed, and clinical symptoms and behavioral alterations were monitored for each recording day from day 0 to day 21 postchallenge.

### Measurement of viral burden in mice.

Subsets of mice were euthanized, and serum, brain, kidney, spleen, and lung samples were dissected. Organs were weighed and homogenized by a bead-beater apparatus. Total RNA was extracted from samples using the RNeasy kit according to the manufacturer’s protocol (Qiagen). Viral load was determined by qRT-PCR as previously described (47). The levels of viral RNA were expressed on a log_10_ scale as viral RNA copies per mL or per mg tissue after comparison with a standard curve produced using serial 10-fold dilutions of TBEV RNA.

### Data availability.

The data that support the findings of this study are available within this article and its supplemental materials. Additional data related to this paper may be requested from the authors.

### Statistical analyses.

Graphical representation and statistical analyses were performed using SigmaPlot 10.0 (Stystat Software) and Origin 8.0 (Origin Software). Results are shown as means ± standard deviation. Unpaired *t* tests were used as indicated for two-group comparisons. A one-way analysis of variance (ANOVA) was used when the study included more than two groups. Differences were considered statistically significant when the *P* value was <0.05 (*, *P* < 0.05).

## References

[1] Gould EA, Solomon T. 2008. Pathogenic flaviviruses. Lancet 371:500–509. doi:10.1016/S0140-6736(08)60238-X. 18262042

[2] Lindquist L, Vapalahti O. 2008. Tick-borne encephalitis. Lancet 371:1861–1871. doi:10.1016/S0140-6736(08)60800-4. 18514730

[3] Velay A, Paz M, Cesbron M, Gantner P, Solis M, Soulier E, Argemi X, Martinot M, Hansmann Y, Fafi-Kremer S. 2019. Tick-borne encephalitis virus: molecular determinants of neuropathogenesis of an emerging pathogen. Crit Rev Microbiol 45:472–493. doi:10.1080/1040841X.2019.1629872. 31267816

[4] Kunze U. 2011. Tick-borne encephalitis: the impact of epidemiology, changing lifestyle, and environmental factors. Conference report of the 12th Annual Meeting of the International Scientific Working Group on Tick-Borne Encephalitis (ISW-TBE). Vaccine 29:1355–1356. doi:10.1016/j.vaccine.2010.12.048. 21199705

[5] Suss J. 2008. Tick-borne encephalitis in Europe and beyond–the epidemiological situation as of 2007. Euro Surveill 13:18916. https://www.eurosurveillance.org/content/10.2807/ese.13.26.18916-en. 18761916

[6] Mukhopadhyay S, Kuhn RJ, Rossmann MG. 2005. A structural perspective of the flavivirus life cycle. Nat Rev Microbiol 3:13–22. doi:10.1038/nrmicro1067. 15608696

[7] Kroschewski H, Allison SL, Heinz FX, Mandl CW. 2003. Role of heparan sulfate for attachment and entry of tick-borne encephalitis virus. Virology 308:92–100. doi:10.1016/s0042-6822(02)00097-1. 12706093

[8] Kopecký J, Grubhoffer L, Kovár V, Jindrák L, Vokurková D. 1999. A putative host cell receptor for tick-borne encephalitis virus identified by anti-idiotypic antibodies and virus affinoblotting. Intervirology 42:9–16. doi:10.1159/000024954. 10393498

[9] Zhang X, Zheng Z, Liu X, Shu B, Mao P, Bai B, Hu Q, Luo M, Ma X, Cui Z, Wang H. 2016. Tick-borne encephalitis virus induces chemokine RANTES expression via activation of IRF-3 pathway. J Neuroinflammation 13:209. doi:10.1186/s12974-016-0665-9. 27576490 PMC5004318

[10] Ichimura T, Asseldonk EJ, Humphreys BD, Gunaratnam L, Duffield JS, Bonventre JV. 2008. Kidney injury molecule-1 is a phosphatidylserine receptor that confers a phagocytic phenotype on epithelial cells. J Clin Invest 118:1657–1668. doi:10.1172/JCI34487. 18414680 PMC2293335

[11] Amara A, Mercer J. 2015. Viral apoptotic mimicry. Nat Rev Microbiol 13:461–469. doi:10.1038/nrmicro3469. 26052667 PMC7097103

[12] Jemielity S, Wang JJ, Chan YK, Ahmed AA, Li W, Monahan S, Bu X, Farzan M, Freeman GJ, Umetsu DT, Dekruyff RH, Choe H. 2013. TIM-family proteins promote infection of multiple enveloped viruses through virion-associated phosphatidylserine. PLoS Pathog 9:e1003232. doi:10.1371/journal.ppat.1003232. 23555248 PMC3610696

[13] Moller-Tank S, Kondratowicz AS, Davey RA, Rennert PD, Maury W. 2013. Role of the phosphatidylserine receptor TIM-1 in enveloped-virus entry. J Virol 87:8327–8341. doi:10.1128/JVI.01025-13. 23698310 PMC3719829

[14] Kondratowicz AS, Lennemann NJ, Sinn PL, Davey RA, Hunt CL, Moller-Tank S, Meyerholz DK, Rennert P, Mullins RF, Brindley M, Sandersfeld LM, Quinn K, Weller M, McCray PB Jr, Chiorini J, Maury W. 2011. T-cell immunoglobulin and mucin domain 1 (TIM-1) is a receptor for Zaire Ebolavirus and Lake Victoria Marburgvirus. Proc Natl Acad Sci USA 108:8426–8431. doi:10.1073/pnas.1019030108. 21536871 PMC3100998

[15] Mercer J, Helenius A. 2008. Vaccinia virus uses macropinocytosis and apoptotic mimicry to enter host cells. Science 320:531–535. doi:10.1126/science.1155164. 18436786

[16] Soares MM, King SW, Thorpe PE. 2008. Targeting inside-out phosphatidylserine as a therapeutic strategy for viral diseases. Nat Med 14:1357–1362. doi:10.1038/nm.1885. 19029986 PMC2597367

[17] Meertens L, Carnec X, Lecoin MP, Ramdasi R, Guivel-Benhassine F, Lew E, Lemke G, Schwartz O, Amara A. 2012. The TIM and TAM families of phosphatidylserine receptors mediate dengue virus entry. Cell Host Microbe 12:544–557. doi:10.1016/j.chom.2012.08.009. 23084921 PMC3572209

[18] Michelitsch A, Fast C, Sick F, Tews BA, Stiasny K, Bestehorn-Willmann M, Dobler G, Beer M, Wernike K. 2021. Long-term presence of tick-borne encephalitis virus in experimentally infected bank voles (Myodes glareolus). Ticks Tick Borne Dis 12:101693. doi:10.1016/j.ttbdis.2021.101693. 33690089

[19] Nagy A, Nagy O, Tarcsai K, Farkas Á, Takács M. 2018. First detection of tick-borne encephalitis virus RNA in clinical specimens of acutely ill patients in Hungary. Ticks Tick Borne Dis 9:485–489. doi:10.1016/j.ttbdis.2017.12.017. 29373305

[20] Caracciolo I, Bassetti M, Paladini G, Luzzati R, Santon D, Merelli M, Sabbata GD, Carletti T, Marcello A, D’Agaro P. 2015. Persistent viremia and urine shedding of tick-borne encephalitis virus in an infected immunosuppressed patient from a new epidemic cluster in North-Eastern Italy. J Clin Virol 69:48–51. doi:10.1016/j.jcv.2015.05.019. 26209378

[21] Lazear HM, Govero J, Smith AM, Platt DJ, Fernandez E, Miner JJ, Diamond MS. 2016. A Mouse model of Zika virus pathogenesis. Cell Host Microbe 19:720–730. doi:10.1016/j.chom.2016.03.010. 27066744 PMC4866885

[22] Samuel MA, Diamond MS. 2005. Alpha/beta interferon protects against lethal West Nile virus infection by restricting cellular tropism and enhancing neuronal survival. J Virol 79:13350–13361. doi:10.1128/JVI.79.21.13350-13361.2005. 16227257 PMC1262587

[23] Füzik T, Formanová P, Růžek D, Yoshii K, Niedrig M, Plevka P. 2018. Structure of tick-borne encephalitis virus and its neutralization by a monoclonal antibody. Nat Commun 9:436. doi:10.1038/s41467-018-02882-0. 29382836 PMC5789857

[24] Moore JP, Doms RW. 2003. The entry of entry inhibitors: a fusion of science and medicine. Proc Natl Acad Sci USA 100:10598–10602. doi:10.1073/pnas.1932511100. 12960367 PMC196849

[25] Dejarnac O, Hafirassou ML, Chazal M, Versapuech M, Gaillard J, Perera-Lecoin M, Umana-Diaz C, Bonnet-Madin L, Carnec X, Tinevez JY, Delaugerre C, Schwartz O, Roingeard P, Jouvenet N, Berlioz-Torrent C, Meertens L, Amara A. 2018. TIM-1 ubiquitination mediates dengue virus entry. Cell Rep 23:1779–1793. doi:10.1016/j.celrep.2018.04.013. 29742433

[26] Wang F, He W, Yuan J, Wu K, Zhou H, Zhang W, Chen ZK. 2008. Activation of Tim-3-Galectin-9 pathway improves survival of fully allogeneic skin grafts. Transpl Immunol 19:12–19. doi:10.1016/j.trim.2008.01.008. 18346632

[27] Chiou B, Lucassen E, Sather M, Kallianpur A, Connor J. 2018. Semaphorin4A and H-ferritin utilize Tim-1 on human oligodendrocytes: a novel neuro-immune axis. Glia 66:1317–1330. doi:10.1002/glia.23313. 29457657 PMC7009020

[28] Yang L, Brooks CR, Xiao S, Sabbisetti V, Yeung MY, Hsiao LL, Ichimura T, Kuchroo V, Bonventre JV. 2015. KIM-1-mediated phagocytosis reduces acute injury to the kidney. J Clin Invest 125:1620–1636. doi:10.1172/JCI75417. 25751064 PMC4396492

[29] Thomas LJ, Vitale L, O’Neill T, Dolnick RY, Wallace PK, Minderman H, Gergel LE, Forsberg EM, Boyer JM, Storey JR, Pilsmaker CD, Hammond RA, Widger J, Sundarapandiyan K, Crocker A, Marsh HC Jr, Keler T. 2016. Development of a novel antibody-drug conjugate for the potential treatment of ovarian, lung, and renal cell carcinoma expressing TIM-1. Mol Cancer Ther 15:2946–2954. doi:10.1158/1535-7163.MCT-16-0393. 27671527

[30] Younan P, Iampietro M, Nishida A, Ramanathan P, Santos RI, Dutta M, Lubaki NM, Koup RA, Katze MG, Bukreyev A. 2017. Ebola virus binding to Tim-1 on T lymphocytes induces a cytokine storm. mBio 8:00845-17. doi:10.1128/mBio.00845-17. PMC5615193 28951472

[31] Brunton B, Rogers K, Phillips EK, Brouillette RB, Bouls R, Butler NS, Maury W. 2019. TIM-1 serves as a receptor for Ebola virus in vivo, enhancing viremia and pathogenesis. PLoS Negl Trop Dis 13:e0006983. doi:10.1371/journal.pntd.0006983. 31242184 PMC6615641

[32] Zhang X, Zheng Z, Shu B, Mao P, Bai B, Hu Q, Cui Z, Wang H. 2016. Isolation and characterization of a Far-Eastern strain of tick-borne encephalitis virus in China. Virus Res 213:6–10. doi:10.1016/j.virusres.2015.11.006. 26555163

[33] Yang Q, Pei R, Wang Y, Zhou Y, Yang M, Chen X, Chen J. 2021. ADAM15 participates in tick-borne encephalitis virus replication. J Virol 95:01926-20. doi:10.1128/JVI.01926-20. PMC7851565 33208450

[34] Zhang Q, Tian F, Wang F, Guo Z, Cai M, Xu H, Wang H, Yang G, Shi X, Shan Y, Cui Z. 2020. Entry dynamics of single Ebola virus revealed by force tracing. ACS Nano 14:7046–7054. doi:10.1021/acsnano.0c01739. 32383590

[35] Hu M, Wang F, Li W, Zhang X, Zhang Z, Zhang XE, Cui Z. 2019. Ebola virus uptake into polarized cells from the apical surface. Viruses 11:1117. doi:10.3390/v11121117. PMC6949903 31810353

[36] Qin C, Li W, Li Q, Yin W, Zhang X, Zhang Z, Zhang XE, Cui Z. 2019. Real-time dissection of dynamic uncoating of individual influenza viruses. Proc Natl Acad Sci USA 116:2577–2582. doi:10.1073/pnas.1812632116. 30626642 PMC6377448

[37] Chang JS, Chi SC. 2015. GHSC70 is involved in the cellular entry of nervous necrosis virus. J Virol 89:61–70. doi:10.1128/JVI.02523-14. 25320288 PMC4301162

[38] Tayyari F, Marchant D, Moraes TJ, Duan W, Mastrangelo P, Hegele RG. 2011. Identification of nucleolin as a cellular receptor for human respiratory syncytial virus. Nat Med 17:1132–1135. doi:10.1038/nm.2444. 21841784

[39] Flint M, McMullan LK, Dodd KA, Bird BH, Khristova ML, Nichol ST, Spiropoulou CF. 2014. Inhibitors of the tick-borne, hemorrhagic fever-associated flaviviruses. Antimicrob Agents Chemother 58:3206–3216. doi:10.1128/AAC.02393-14. 24663025 PMC4068503

[40] Li M, Ablan SD, Miao C, Zheng YM, Fuller MS, Rennert PD, Maury W, Johnson MC, Freed EO, Liu SL. 2014. TIM-family proteins inhibit HIV-1 release. Proc Natl Acad Sci USA 111::E3699–E3707. doi:10.1073/pnas.1404851111. 25136083 PMC4156686

[41] Mandl CW, Ecker M, Holzmann H, Kunz C, Heinz FX. 1997. Infectious cDNA clones of tick-borne encephalitis virus European subtype prototypic strain Neudoerfl and high virulence strain Hypr. J Gen Virol 78:1049–1057. doi:10.1099/0022-1317-78-5-1049. 9152422

[42] Zhang X, Zheng Z, Shu B, Liu X, Zhang Z, Liu Y, Bai B, Hu Q, Mao P, Wang H. 2013. Human astrocytic cells support persistent coxsackievirus B3 infection. J Virol 87:12407–12421. doi:10.1128/JVI.02090-13. 24027313 PMC3807905

[43] Volchkov VE, Volchkova VA, Muhlberger E, Kolesnikova LV, Weik M, Dolnik O, Klenk HD. 2001. Recovery of infectious Ebola virus from complementary DNA: RNA editing of the GP gene and viral cytotoxicity. Science 291:1965–1969. doi:10.1126/science.1057269. 11239157

[44] Yu CY, Hsu YW, Liao CL, Lin YL. 2006. Flavivirus infection activates the XBP1 pathway of the unfolded protein response to cope with endoplasmic reticulum stress. J Virol 80:11868–11880. doi:10.1128/JVI.00879-06. 16987981 PMC1642612

[45] Cheng G, Zhong J, Chung J, Chisari FV. 2007. Double-stranded DNA and double-stranded RNA induce a common antiviral signaling pathway in human cells. Proc Natl Acad Sci USA 104:9035–9040. doi:10.1073/pnas.0703285104. 17517627 PMC1885623

[46] Müller U, Steinhoff U, Reis LF, Hemmi S, Pavlovic J, Zinkernagel RM, Aguet M. 1994. Functional role of type I and type II interferons in antiviral defense. Science 264:1918–1921. doi:10.1126/science.8009221. 8009221

[47] Schwaiger M, Cassinotti P. 2003. Development of a quantitative real-time RT-PCR assay with internal control for the laboratory detection of tick borne encephalitis virus (TBEV) RNA. J Clin Virol 27:136–145. doi:10.1016/s1386-6532(02)00168-3. 12829035

