# Supplementary figures and images for: T-Cell Immunoglobulin and Mucin Domain 1 (TIM-1) Is a Functional Entry Factor for Tick-Borne Encephalitis Virus
Source: mBio. 2022 Jan 25;13(1):e02860-21. doi: 10.1128/mbio.02860-21 (PMC8787471; doi:10.1128/mbio.02860-21)

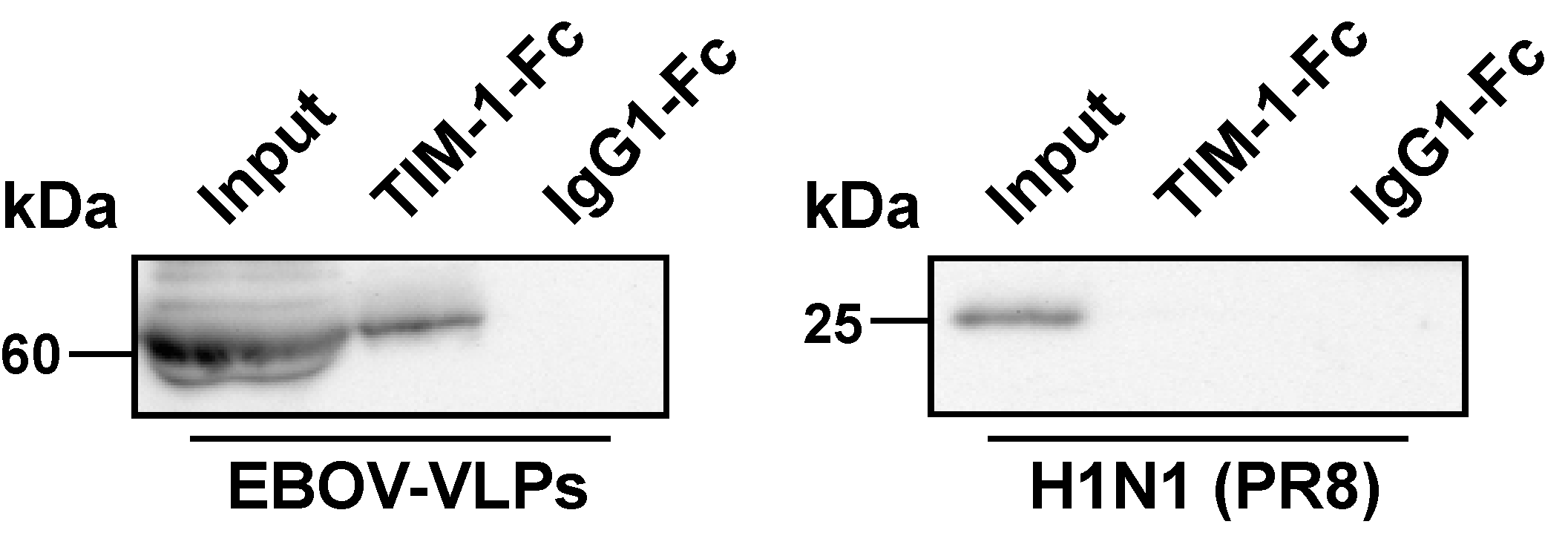

Supplement: FIG S1 [file mbio.02860-21-sf001.tif]

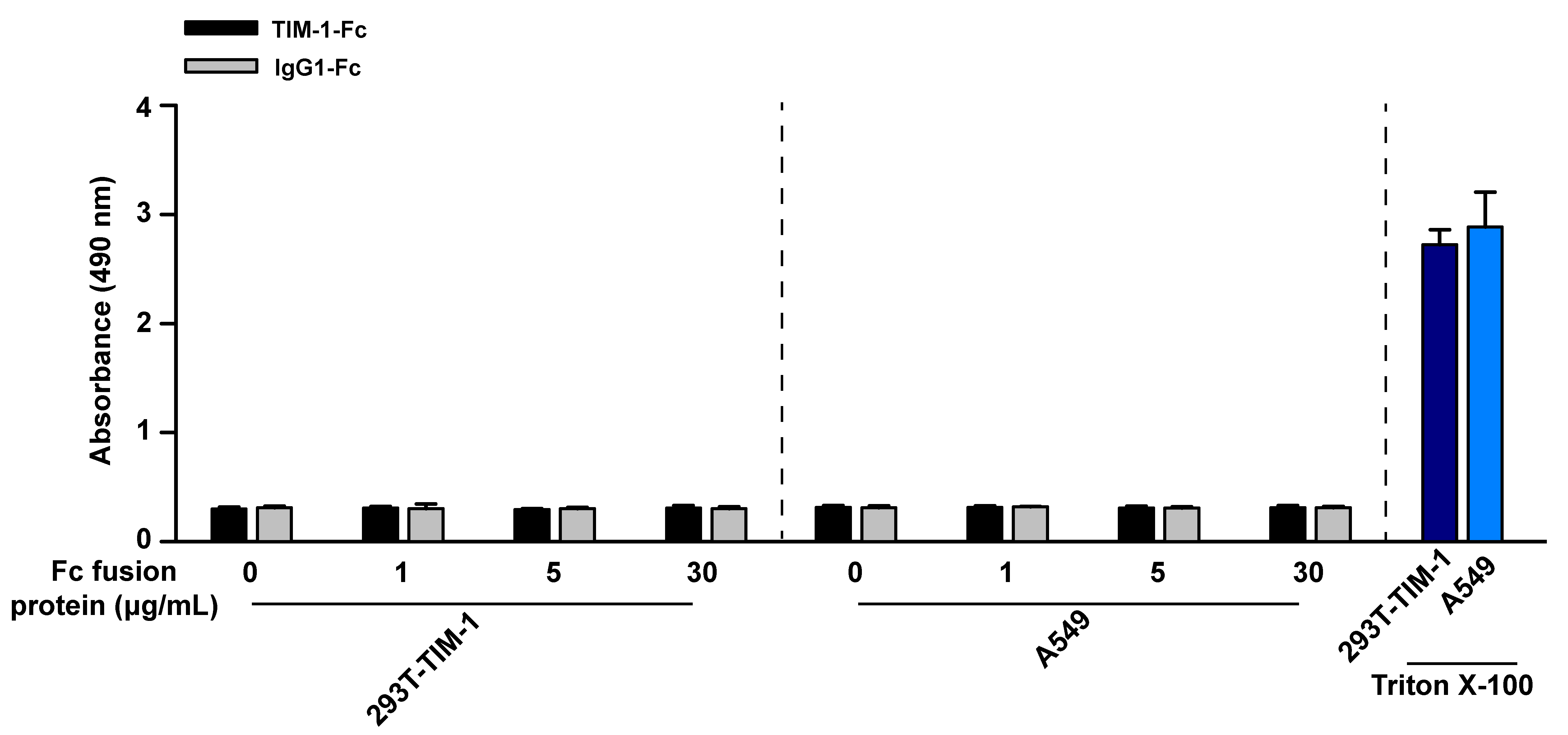

Supplement: FIG S2 [file mbio.02860-21-sf002.tif]

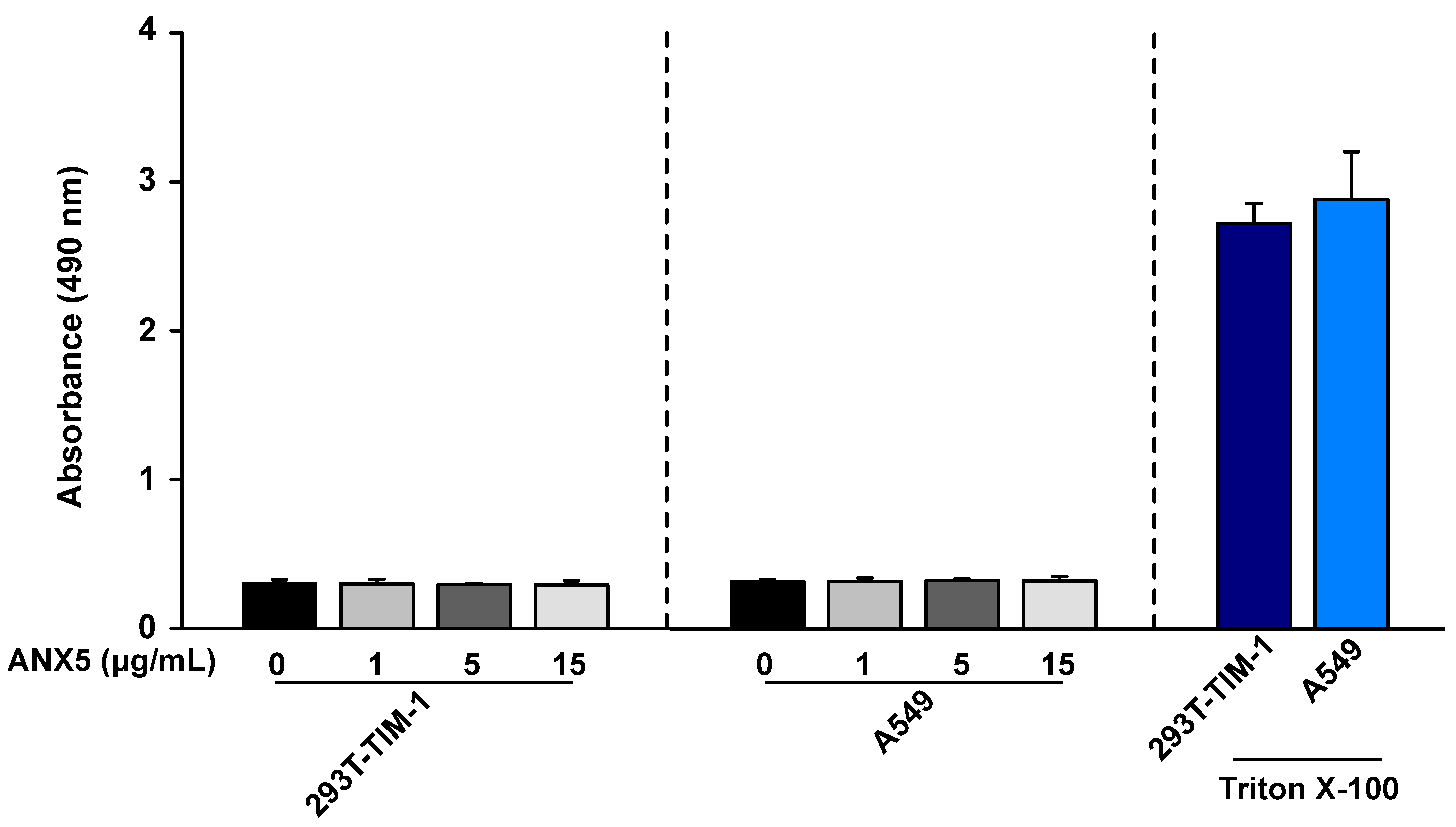

Supplement: FIG S3 [file mbio.02860-21-sf003.tif]

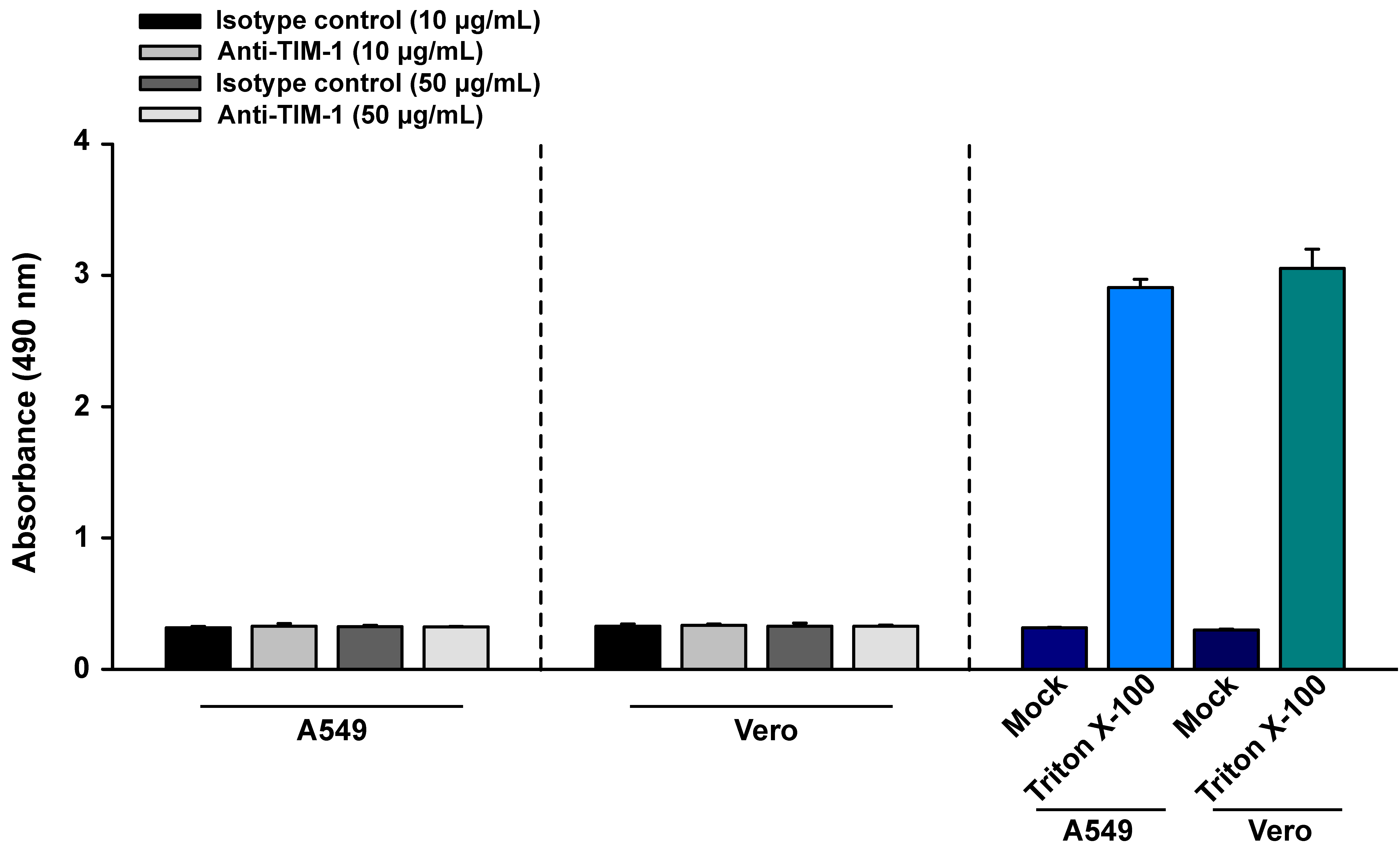

Supplement: FIG S4 [file mbio.02860-21-sf004.tif]

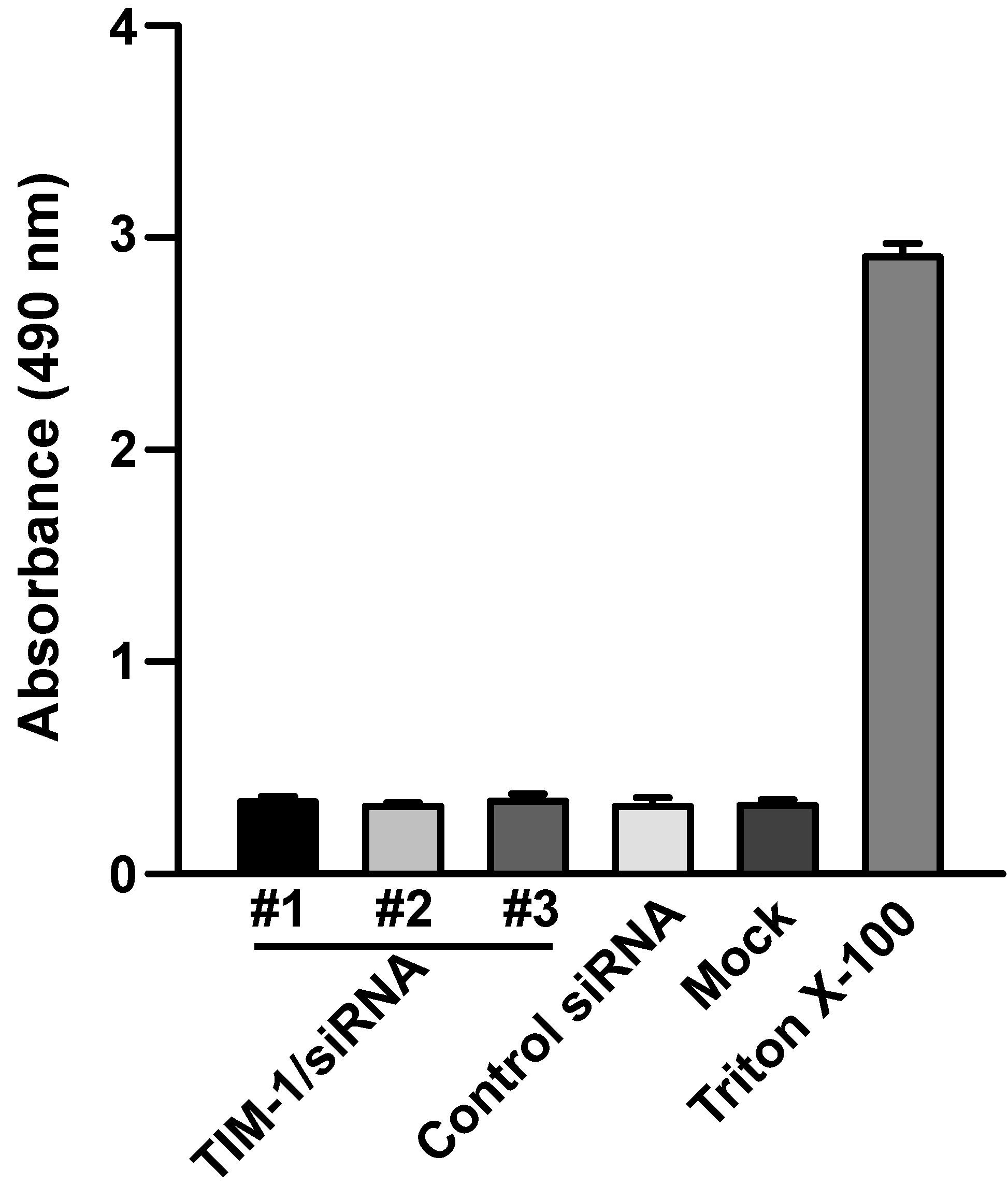

Supplement: FIG S5 [file mbio.02860-21-sf005.tif]

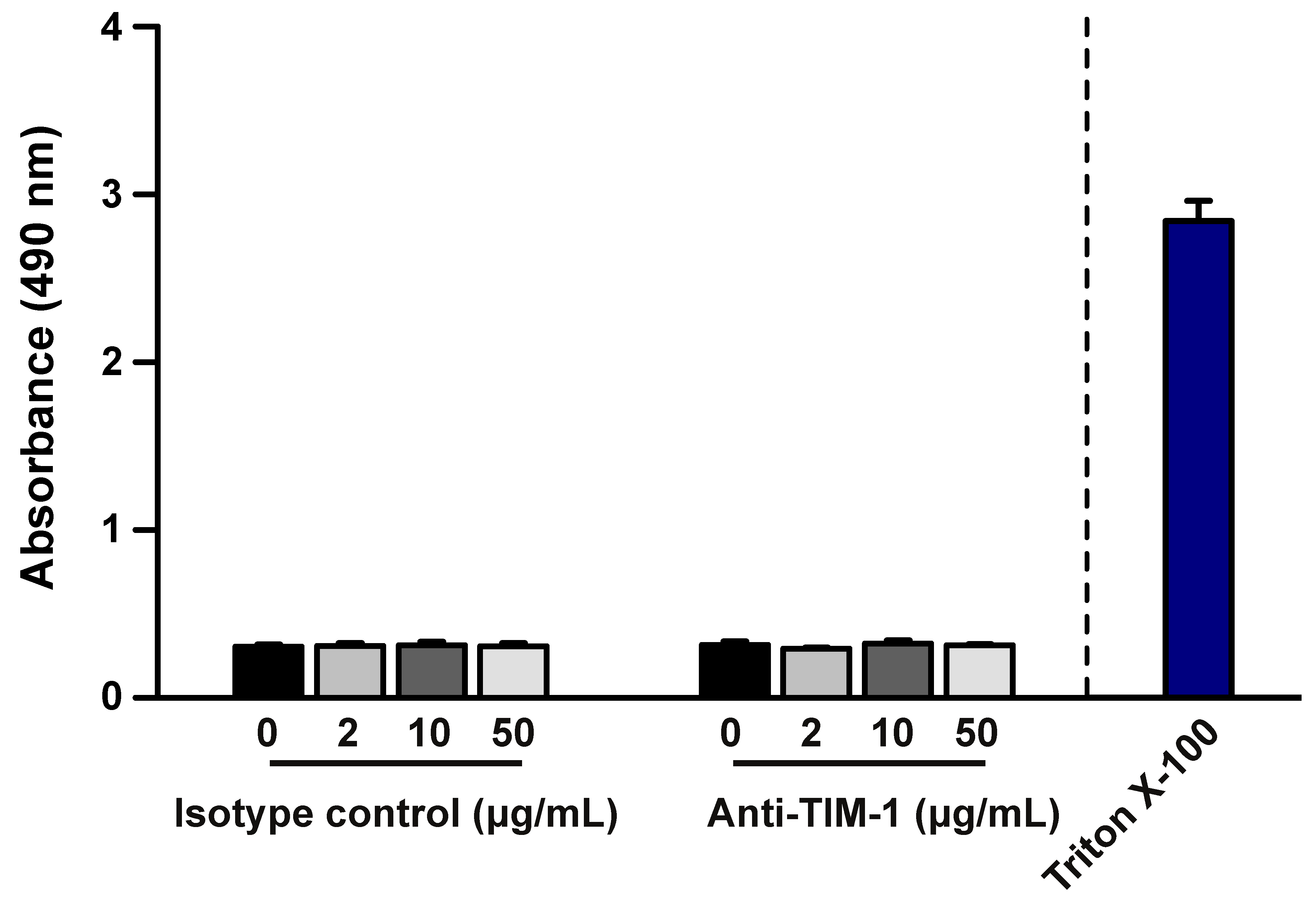

Supplement: FIG S6 [file mbio.02860-21-sf006.tif]

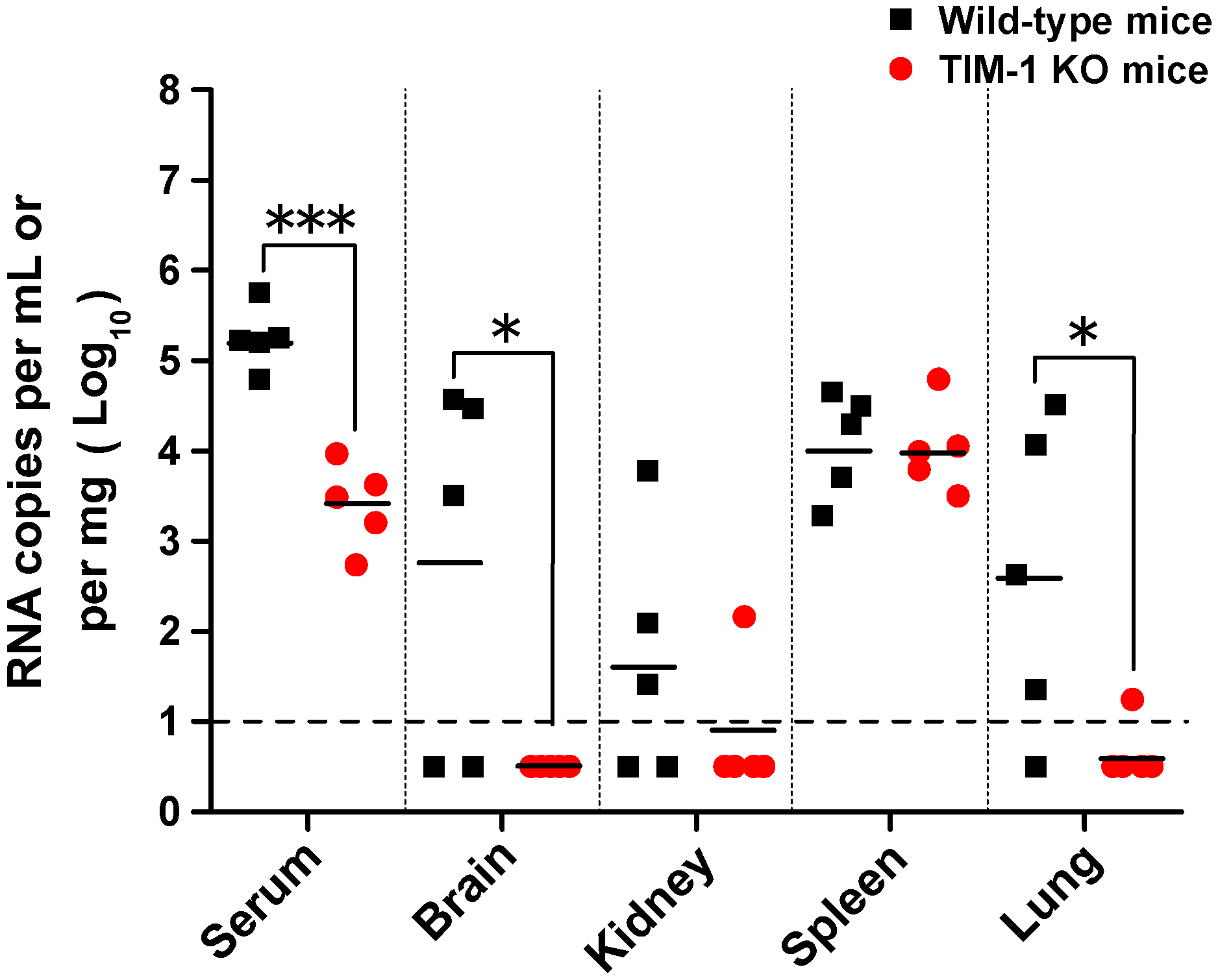

Supplement: FIG S7 [file mbio.02860-21-sf007.tif]
